# Transcriptional Shift Identifies a Set of Genes Driving Breast Cancer Chemoresistance

**DOI:** 10.1371/journal.pone.0053983

**Published:** 2013-01-10

**Authors:** Laura Vera-Ramirez, Pedro Sanchez-Rovira, Cesar L. Ramirez-Tortosa, Jose L. Quiles, MCarmen Ramirez-Tortosa, Jose A. Lorente

**Affiliations:** 1 Department of Oncology, Complejo Hospitalario de Jaen, Jaen, Spain; 2 GENyO Center, Pfizer-University of Granada & Andalusian Government Centre for Genomics & Oncology, Granada, Spain; 3 Department of Pathology, Complejo Hospitalario de Jaen, Jaen, Spain; 4 Institute of Nutrition and Food Technology “José Mataix”, Biomedical Research Center (CIBM), Granada, Spain; 5 Department of Physiology, University of Granada, Granada, Spain; 6 Department of Biochemistry and Molecular Biology II, University of Granada, Granada, Spain; 7 Department of Legal Medicine, University of Granada, Granada, Spain; Baylor College of Medicine, United States of America

## Abstract

**Background:**

Distant recurrences after antineoplastic treatment remain a serious problem for breast cancer clinical management, which threats patients’ life. Systemic therapy is administered to eradicate cancer cells from the organism, both at the site of the primary tumor and at any other potential location. Despite this intervention, a significant proportion of breast cancer patients relapse even many years after their primary tumor has been successfully treated according to current clinical standards, evidencing the existence of a chemoresistant cell subpopulation originating from the primary tumor.

**Methods/Findings:**

To identify key molecules and signaling pathways which drive breast cancer chemoresistance we performed gene expression analysis before and after anthracycline and taxane-based chemotherapy and compared the results between different histopathological response groups (good-, mid- and bad-response), established according to the Miller & Payne grading system. Two cohorts of 33 and 73 breast cancer patients receiving neoadjuvant chemotherapy were recruited for whole-genome expression analysis and validation assay, respectively. Identified genes were subjected to a bioinformatic analysis in order to ascertain the molecular function of the proteins they encode and the signaling in which they participate. High throughput technologies identified 65 gene sequences which were over-expressed in all groups (*P* ≤ 0·05 Bonferroni test). Notably we found that, after chemotherapy, a significant proportion of these genes were over-expressed in the good responders group, making their tumors indistinguishable from those of the bad responders in their expression profile (*P* ≤ 0.05 Benjamini-Hochgerg`s method).

**Conclusions:**

These data identify a set of key molecular pathways selectively up-regulated in post-chemotherapy cancer cells, which may become appropriate targets for the development of future directed therapies against breast cancer.

## Introduction

The searching for predictive tumor biomarkers in breast cancer treatment has been a major research issue for decades [1], [2]. Indeed, the scientific community is conscious that a better and more accurate system to identify individuals at increased risk of recurrence, avoid under- and over-treatment and improve the long-term survival rates of the patients, is needed [3], [4]. In this regard, the revolutionary scientific and technological advances in the field of genomics has allowed the development of multi-gene assays which have been approved and commercialized to guide clinical decisions according with the particular characteristics of tumors [5]-[9]. Despite substantial advances in this field, around 30% of patients with early-stage breast cancer relapse after an unpredictable period, even if they achieved a good response to systemic treatment [3], [5]. This and other scientific findings, such as the detection of circulating tumor cells (CTCs) in the bloodstream of treated patients, has pointed out tumor chemoresistance as a leading process involved in breast cancer progression [10], [11].

Clearly, to be able to overcome cancer chemoresistance we must first acquire a complete understanding of the molecular processes and leading alterations which make possible this effect. In this regard, a novel hypothesis involving dedifferentiated cells with stem-like properties has been highlighted. The cancer stem cell (CSC) hypothesis assumes that some neoplasms, such as breast cancer, are the consequence of the accumulation of transforming genetic and epigenetic changes in adult stem cells or their progressively differentiated progenitors. First experimental evidence was reported by Al-Hajj et al. [12] who showed that the transplantation of few cells characterized by the CD44+/CD24- inmunophenotype in non-obese diabetic/severe combined immunodeficient (NOD/SCID) immunocompromised mice formed tumors that recapitulated the phenotypic heterogeneity of the original breast tumors from which they were derived. Since then, many studies have focused on the study of this putative tumor-initiating cell population [13]. Remarkably, CSCs have been related to chemoresistance since they present a low proliferation rate, over-express cell surface proteins involved in drug efflux and dug-metabolizing enzymes [14]. It has also been reported that CSCs exert a radio-resistant behavior through the over-expression of free radical scavenging systems [15]. Nevertheless, key molecular processes conferring the ability to overcome drug-induced toxicity and promote cancer cell survival in order to establish a new oncogenic lesion, even several years after successful systemic treatment, remains to be elucidated despite an increasing amount of data regarding this issue is published yearly.

In relation to this issue, important concepts has been introduced changing the way that scientist approach to the study of chemoresistance and other oncologic events. From the works of Greenman et al. [16] and Sjøblom et al. [17] it can be extracted that tumors arise from the alteration of key genes known as “diver mutations” which also direct tumor biology. The rest of genes reported to be mutated at a lower frequency across different tumor samples were termed “passenger mutations”, which are thought to be a consequence of tumor genomic instability but have a modest impact on tumor phenotype. Indeed, it has been suggested that the occurrence of passenger mutations and associated gene over- and under-expression may be on the base of confounding results from genomic studies, which may have identified different tumoral/clinical entities under the same genomic category according to the differential expression of genes that are transiently modified due to the highly genomic instability to which are subjected tumor cells [1]. Genes affected by driver mutations, the molecular processes they direct and the signaling pathways in which they participate are of great scientific interest given their therapeutic potential.

To investigate the biological processes which drive chemoresistance in breast cancer, we designed and performed a gene expression study which included patients who registered all possible pathologic responses to this treatment. The comparison of gene expression differences induced by chemotherapy allowed the identification of 30 genes which were over-expressed after chemotherapy regardless patient’s response to treatment.

## Methods

### Ethics statement

This study was approved by the local Ethical Review Board, Comité de Ética de la Investigación de Jaén, and in accordance with Good Clinical Practices and the tenets of the Declaration of Helsinki. Patients provided their written informed consent to participate in this study.

### Patient population

This study included two independent sets of patients involving a total of 118 breast cancer cases. All patients were staged based on physical examination, radiologic findings and pathologic examination of tumor biopsies. Initially, we recruited 46 patients with a histologically confirmed diagnosis of breast cancer and scheduled neoadjuvant chemotherapy treatment based on Anthracyclines and Taxanes, as determined by the medical oncology team. From this cohort, 33 patients met the inclusion criteria for genome-wide expression analysis with oligonucleotide microarrays. Nevertheless, gene expression data from 4 pre-chemotherapy samples and 1 post-chemotherapy sample were discarded from final analysis as microarrays data quality control identified them as outliers. Consequently, whole genome gene expression analysis finally included 56 matched pre- and post-chemotherapy samples from 28 cases plus 4 pre-chemotherapy and 1 post-chemotherapy samples from 5 additional cases. Globally, 33 cases and 61 samples were processed for whole genome expression analysis. To validate the results derived from gene expression analysis, a qRT-PCR assay was designed and formalin fixed paraffin embedded (FFPE) tumor samples from 85 selected breast cancer cases who received equivalent neoadjuvant chemotherapy were processed. Despite 85 patients were initially included in this validation experiment, a final sample size of 73 cases were selected for data analysis due to either of the following reasons: the patient refused chemotherapy, the medical oncologist finally decided to change the treatment scheduled, the patient experienced acute toxicity and did not finish the treatment. The pathologic and clinical information from each patient was extracted from the medical reports achieved in the Oncology Department Registry.

### Tumor tissue samples

Tumor samples from each patient included in this study (Table 1) were obtained before and after chemotherapy. Pre-chemotherapy tumor samples from the initial cohort were obtained during diagnosis through ultrasound-guided core needle biopsy and post-chemotherapy tumor samples were obtained from surgery pieces after mastectomy or surgical resection. Two core needle biopsies were obtained from each of the cases included, one was flash frozen in liquid nitrogen and stored at -80°C and the other was fixed in buffered formalin and embedded in paraffin for standard histological and immunohistochemical analyses. The same procedure was followed to process and store the post-chemotherapy tumor samples. Pre- and post-chemotherapy tumor samples were frozen within 30 min after biopsy or surgery. Notably, this assay was not biased for individual genetic differences, as most samples used corresponded to paired pre- and post-treatment samples from each case. Breast cancer patients were distributed in experimental groups according to their pathological responses to anthraclycline and taxane-based chemotherapy, as determined by the Miller and Payne grading system [18]. This resulted in a group of good responders -GR (Miller & Payne grades 4 and 5)-, a group of mid response –MR (Miller & Payne grade 3)- and a group of bad responders -BR (Miller & Payne grades 1 and 2)-. For whole-genome expression analysis cases were selected irrespectively of their Human Epidermal growth factor Receptor 2 (Her2) status, unless it was registered as a phenotypic tumor characteristic. Validation experiment was performed in an independent set of 170 paired formalin fixed paraffin embedded (FFPE) samples (before and after chemotherapy) corresponding to 85 breast cancer cases. The validation cohort comprised all pathological response groups described and an extra group of patients with Human Epidermal growth factor Receptor 2 (Her2) positive tumors (Her2G). Her2-positive tumors are very different entities from the molecular point of view [19]. On the other hand, the addition of Trastuzumab, a therapeutic monoclonal antibody against Her2, to anthraclycline and taxane-based chemotherapy is routinely scheduled for these patients as it greatly improves their pathological response to treatment [20]. Then, the molecular and clinical distinctive characteristics of Her2-positive tumors were the main arguments to include this extra group in our experimental design. Additionally, the MR group was further subdivided into two groups for validation analysis –mid-response high (MRH) and mid-response low (MRL)-. As in the case of whole genome expression assay and for the same reasons, this study was not biased for individual genetic differences among cases (see Supporting Methods (Text S1) and Figure S1 for a detailed explanation of the procedures followed for tumor tissue selection).

**Table 1:** Population demographics and pre-chemotherapy clinical characteristics.

	Whole-genome expression analysis cohort	Validation assay cohort
**Age at diagnosis in years**		
< 40	5 (15.1)	10 (13.7)
40-49	9 (27.3)	30 (41.1)
50-59	10 (30.3)	11 (15.1)
≥ 60	9 (27.3)	22 (30.1)
Median	54	50.45
Range	29-74	26-75
**Histological type**		
Ductal	30 (90.9)	65 (89.1)
Lobular	3 (3.1)	6 (8.2)
Mixed	0 (0)	2 (2.7)
**T staging**		
T1	0 (0)	2 (2.7)
T2	28 (84.9)	48 (65.8)
T3	4 (12.1)	18 (24.7)
T4	1 (3)	5 (6.8)
**N staging**		
N0	9 (27.3)	38 (52.1)
N1-3	24 (72.7)	35 (47.9)
**AJCC Staging**		
IA/IB	0 (0)	1 (1.4)
IIA/IIB	18 (54.5)	56 (76.7)
IIIA/IIIB/IIIC	15 (45.5)	16 (21.9)
**Bloom-Richardson’s histological grade**		
I	3 (9.1)	22 (30.1)
II	14 (42.4)	28 (38.4)
III	16 (48.5)	23 (31.5)
**Estrogen receptor**		
+	24 (72.7)	55 (75.3)
-	9 (27.3)	18 (26.7)
**Progesterone receptor**		
+	24 (72.7)	42 (57.5)
-	9 (27.3)	31 (42.5)
**Her2**		
+	6 (18.2)	15 (20.5)
-	27 (81.8)	58 (79.5)
**Miller & Payne grade**		
1 and 2 (BR)	15 (45.4)	12 (20.7)
3 (MR)	9 (27.3)	NA
3 – MRL (30-60% tumor cell reduction)-	NA	12 (20.7)
3 – MRH (61-90% tumor cell reduction)-	NA	15 (25.8)
4 and 5 (GR)	9 (27.3)	19 (32.8)

Results are presented as n (%) of 33 patients for the whole-genome expression analysis cohort and as n (%) of 73 patients for the validation assay cohort.

Abbreviations: AJCC, American Joint Committee on Cancer; BR, bad response group; GR, good response group; Her2G, Her2-positive group; MRH, mid-response high group; MRL, mid-response low group; NA, not applicable.

### RNA isolation and microarray analysis

Sample processing, microarray hybridization and gene expression analysis were carried out with the Affymetrix Genechip System (Affymetrix, Santa Clara, CA, USA). Briefly, total RNA was extracted and purified for microarray analysis using QIAshredder columns and the RNeasy Mini kit (Qiagen, Hilden, Germany) according to manufacturer’s instructions. From 1μg of total RNA, complementary DNA (cDNA) was synthesized using the One-Cycle cDNA Synthesis kit (Affymetrix). Biotinylated complementary RNA (cRNA) was synthesized following the IVT labeling kit (Affymetrix) and purified using the GeneChip Sample Cleanup Module (Affymetrix). Subsequently, Biotinylated cRNA was fragmented and hybridized to the Genechip Human Genome U133 Plus 2.0 microarrays (Affymetrix). After hybridization, microarrays followed washing and staining protocol and were scanned using the GeneChip Scanner 3000 (Affymetrix). The fluorescent signal corresponding to the intensity of hybridization intensity of each transcript was determined using the Gene Chip Operating Software (GCOS 1.4; Affymetrix). Intensity values were scaled such that the overall fluorescence intensity of each array was equivalent. Finally, probe set measurements were generated from quantified Affymetrix image (.CEL) files using the Robust Multichip Average method (RMA) from the Affy package Bioconductor (available at http://www.bioconductor.org).

Bonferroni test was estimated to correct for multiple tests, considering *P* ≤ 0.05 to be significant, and Fold-change (FC) values were calculated for all comparisons. After non-supervised Principal components analysis (PCA) and clustering, gene expression statistical significances were identified by two linear regression models taking into account the pathologic response to chemotherapy, if the sample was obtained before or after systemic treatment and the matching of pre- and post-chemotherapy samples derived from the same patient. Supervised PCA analysis and clustering were performed with processed data. Partek Genomics Suite v7.3.1 (Partek, St. Louise, MO, USA) software was employed for the statistic analysis and clustering and the Euclidean distance for similarity measurements, and average linkage was selected as association. Functional enrichment analysis was carried out using the Protein ANalysis THrough Evolutionary Relationships (PANTHER) (http://www.pantherdb.org) and the Database for Annotation, Visualization and Integrated Discovery (DAVID) (http://david.abcc.ncifcrf.gov/) software.

### Quantitative real-time RT-PCR analysis

Based on the microarray results, expression levels of 90 genes were evaluated using qPCR analysis. With this purpose, 5 sections of 10 µm from each formalin-fixed, paraffin-embedded breast cancer sample were processed to isolate 10 ng of total RNA using the RNesay FFPE Kit (Qiagen). cDNAs were reverse-transcribed from total RNA samples using the High Capacity cDNA Archive Kit (Applied Biosystems) according to manufacturer’s instructions. TaqMan PCR reactions were performed on cDNA samples using the Taqman Gene Expression Master Mix (Applied Biosystems) in conjunction with custom 7,900 microfluidic cards (Applied Biosystems) and ABI PRISM 7,900 HT Sequence Detection Systems, according to manufacturer’s instructions. The gene set contained 6 housekeeping genes, GADPH, HPRT1, MRPL19, RPLP0, TBP and TFRC selected according to specific bibliography [21]. According to Genorm calculations, we considered all six housekeeping genes for normalization. Absolute threshold cycle values (Ct values) were determined by using SDS 2.2.2 software (Applied Biosystems).

DCt values were used as dependent variables in the statistical analysis. A linear regression model (Limma) was used to detect differentially expressed changes among groups and Benjamini and Hochberg’s method was used to control the false discovery rate (FDR) with an adjusted *P*-value threshold of 0.05.

Functional networks and pathways analyses were generated through the use of Ingenuity Pathways Analysis (IPA) (Ingenuity Systems®, www.ingenuity.com). Fischer‘s exact test was used to calculate a *P*-value determining the probability that each biological function and/or disease assigned to that dataset and network is due to chance alone (*P*-values ≤ 0.05 were considered as significant). Activation z-score was calculated as a measure of functional and translational activation in Networks and Upstream regulators analysis. An absolute z-score of below (inhibited) or above (activated) 2 was considered as significant (see Supporting Methods (Text S1) for details).

## Results

**Figure 1:**
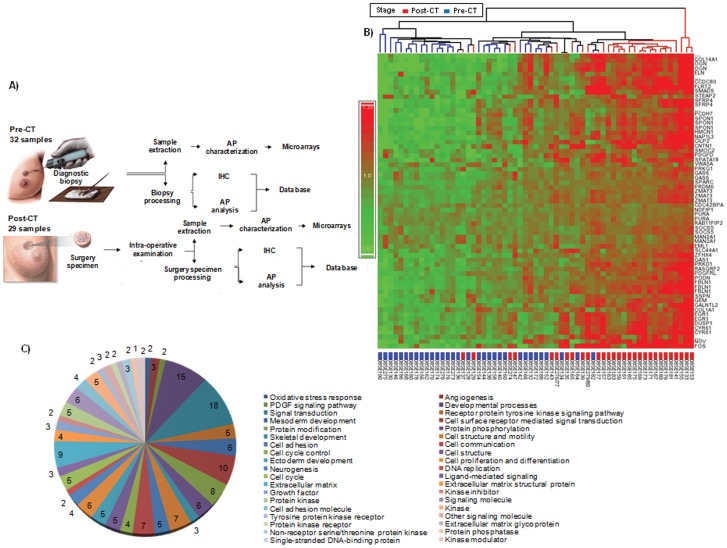
Experimental design and main results from genome-wide expression analysis. A) Experimental design of the discovery assay. B) Genes differentially over-expressed after chemotherapy C) Gene Ontology (GO) terms over-represented by the genes differentially over-expressed after chemotherapy at a significance level of *P*<0.05. Circular representation must be read clockwise and legend must be read from left to right and top to bottom. Numbers within the figure correspond to the number of genes classified in each GO category.

After comparing pre-chemotherapy and post-chemotherapy samples, we identified a subset of 65 gene sequences whose expression was significantly up-regulated after chemotherapy considering all groups (Figure 1A, 1B, Table S1). Gene ontology (GO) analysis revealed that the proteins coded by these genes were mainly involved in extracellular matrix metabolism, cell proliferation and adhesion, oxidative stress response, angiogenesis and developmental processes (Fig. 1C). These are key processes in breast cancer chemoresistance and progression given their central role in invasion and connections with cellular dedifferentiation [2], [22]. Further study of the molecular signaling pathways in which these proteins are involved allowed the selection of 41 out of the 65 gene sequences detected and 49 additional genes of interest to test in a validation assay (Table S2). Remarkably, validation of the microarray results by quantitative reverse transcriptase PCR (qRT-PCR) in an independent data set confirmed previous observations, except for 4 out of the 41 genes selected for validation –CDC42 binding protein kinase alpha (DMPK-like) (CDC42BPA), protocadherin 7 (PCDH7), purine-rich element binding protein A (PURA) and SPARC related modular calcium binding 2 (SMOC2)-. In sum, in a cohort of 106 breast cancer patients and 207 tumor samples we identified a set of 37 genes significantly up-regulated after chemotherapy.

**Figure 2:**
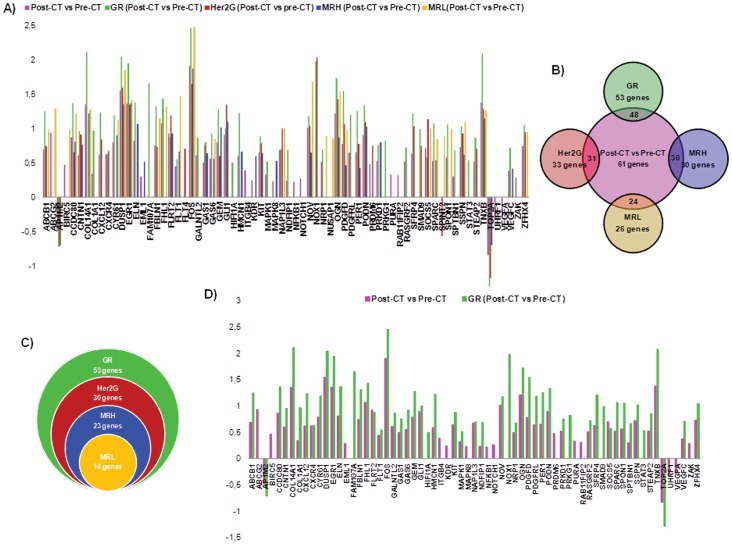
Main results from Post-CT vs Pre-CT comparisons in the validation assay. A) Log_10_ fold change in mRNA abundance of each differentially expressed gene after chemotherapy considering all experimental groups together (Post-CT vs Pre-CT) and each experimental group individually –GR (Post-CT vs Pre-CT), Her2G (Post-CT vs Pre-CT), MRH (Post-CT vs Pre-CT) and MRL (Post-CT vs Pre-CT)-. B) Venn diagram outlining differentially expressed genes after chemotherapy in each pathological response group with respect differentially expressed genes after chemotherapy considering all experimental groups. C) Venn diagram outlining differentially expressed genes after chemotherapy in the four pathological response groups. D) Log_10_ fold change in mRNA abundance of genes differentially expressed after chemotherapy considering all experimental groups together (Post-CT vs Pre-CT) and GR –GR (Post-CT vs Pre-CT)-.

Notably, intra-group differences revealed an increasing number of differentially over-expressed genes after chemotherapy as pathological response to chemotherapy improved (Figure 2A, B, C, Table S3). Post-chemotherapy vs Pre-chemotherapy comparison yielded 55 differentially expressed genes in the GR group, while the same comparison within the BR group did not identify any differentially expressed gene after chemotherapy. Interestingly, all these genes were over-expressed after chemotherapy except two of them -adaptor-related protein complex 1, mu 2 subunit (AP1M2) and topoisomerase (DNA) II alpha (TOP2A)- (Table S4). These results suggest that the high proportion of chemotherapy-induced cancer cell death observed in the GR group is accompanied by pronounced changes in gene expression, while a moderate effect of chemotherapy on cancer cell survival, as noted by microscopic analysis of the pre- and post-chemotherapy samples in the BR group, is also undetectable at the genetic level.

**Figure 3:**
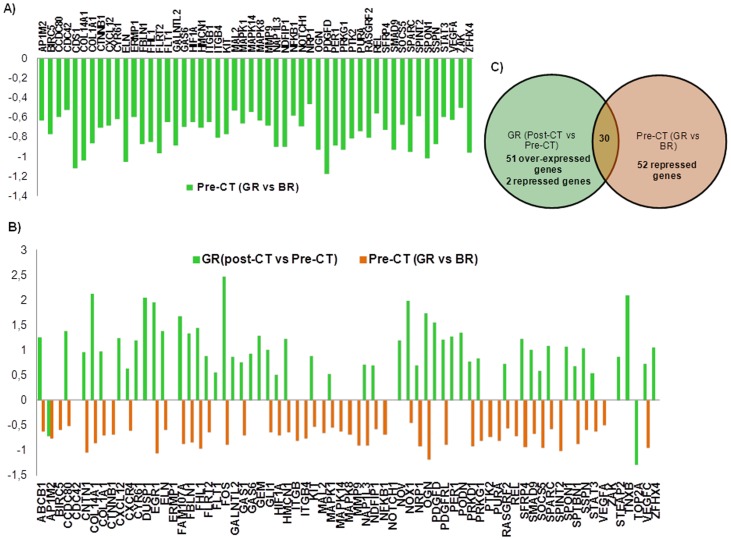
Chemoresistance gene set. A) Log_10_ fold change in mRNA abundance of genes differentially expressed before chemotherapy when comparing GR and BR groups. B) Log_10_ fold change in mRNA abundance of genes differentially expressed for GR (Post-CT vs Pre-CT) comparison and Pre-CT (GR vs BR) comparison C) Venn diagram outlining differentially expressed genes in GR (Post-CT vs Pre-CT) and Pre-CT (GR vs BR) comparisons.

Comparisons between groups before and after chemotherapy yielded interesting and clarifying results. Before chemotherapy, GR and BR groups significantly differed in the expression of 46 sequences, all of which resulted to be repressed in the GR group with respect the BR group (Figure 3A, Table S5). Surprisingly, we realized that 30 of the genes composing the result list of the intra-group comparison for GR group were also present in the comparison GR vs BR before chemotherapy, but with shifted expression, except for AP1M2 (Figure 3B, 3C, Table 2). In other words, we observed that the expression levels of 30 genes, which significantly differed between chemo-sensitive and chemoresistant breast tumors before chemotherapy, shifted from repression to over-expression along chemotherapy in chemo-sensitive tumors. The comparison between GR and BR groups after chemotherapy showed no differences in the expression of any of the genes analyzed, further sustaining this finding.

**Table 2:** Genes differentially over-expressed after chemotherapy within the GR group –GR (Post-CT vs Pre-CT) comparison- and differentially repressed before chemotherapy in the GR group with respect the BR group – Pre-CT (GR vs BR) comparison.

Gene	RQ_ GR (Post-CT vs Pre-CT)_	RQ_ Pre-CT (GR vs BR)_
AP1M2	0.18527341	0.25512521
CCDC80-	24.9035562	0.22079355
CDC42	3.26993026	0.20638594
COL1A1	9.42556898	0.12550981
CTNNB1-	3.16379687	0.19267806
CXCL12	17.7098848	0.18718921
ELN	32.8078197	0.05031032
FBLN1	21.7898132	0.12001847
FLRT2	8.25765462	0.08162272
FLT1	3.66768961	0.22111543
GAS6	9.25756696	0.18383045
HIF1A	3.23169083	0.1967843
HMCN1	17.8855569	0.17215669
KIT	7.21497578	0.18731882
MAPK1	3.32065324	0.21790637
NAP1L3	5.25859157	0.1153033
NDFIP1	5.03897534	0.11807199
OGN	74.4143783	0.07692849
PDGFD	35.7791181	0.0488577
PER1	18.8005316	0.10464691
PRKG1	6.87755998	0.10094629
RASGRF2	5.19824073	0.17215156
SFRP4	16.3994467	0.16618226
SMAD9	9.43966808	0.06364068
SOCS5	3.95622867	0.2120314
SPARC	12.0367405	0.09934201
SPON1	11.6892213	0.07514114
SSPN	11.4258243	0.10431547
ZAK	3.30297386	0.23327466
ZFHX4	11.9025459	0.09150727

RQ _GR (Post-CT vs Pre-CT)_ describes the magnitude of change of each target gene after chemotherapy with respect its expression before chemotherapy for GR group and RQ _Pre-CT (GR vs BR)_ describes the magnitude of change of each target gene in the GR group with respect the BR group before chemotherapy. BR, bad response group; GR, good response group; Post-CT, after chemotherapy; Pre-CT, before chemotherapy; RQ, relative quantity.

**Figure 4:**
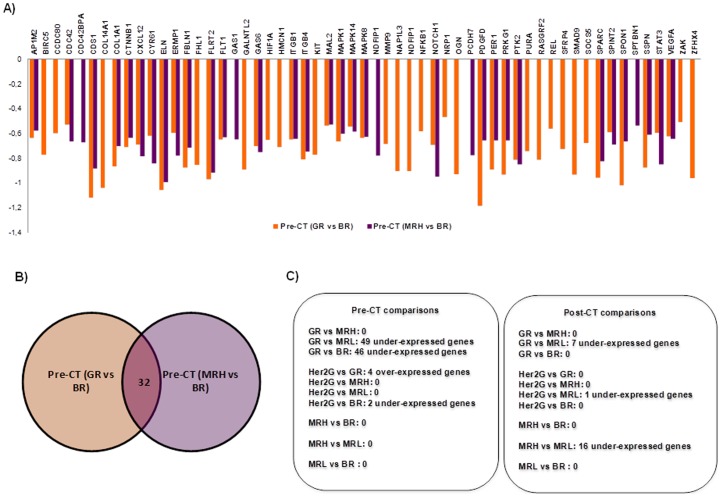
Diagram of pre-chemotherapy and post-chemotherapy comparisons and involvement of mid-response groups. A) Log_10_ fold change in mRNA abundance of genes differentially expressed in common for Pre-CT (GR vs BR) and Pre-CT (GR vs MRL) comparisons. B) Venn diagram outlining differentially expressed genes in common between Pre-CT (GR vs BR) and Pre-CT (GR vs MRL) comparisons. C) Summary of pre-CT and post-CT comparisons.

The comparisons concerning mid-response sub-groups resulted in a variable number of differentially expressed genes highlighting the molecular heterogeneity of intermediate responses to chemotherapy (Table S6, Table S7). Nevertheless, closer results from the comparisons involving MRH and MRL to the results of equivalent comparisons involving GR and BR, respectively, suggest a reliable concordance between histopathological response and the expression of this set of genes (Figure 4). With respect to Her2G, pre-chemotherapy comparisons confirmed the known molecular differences between Her2-positive and Her2-negative tumors but, interestingly, the results from post-chemotherapy comparisons involving Her2G and post-chemotherapy vs pre-chemotherapy comparison within the Her2G showed a higher degree of homology with those involving GR tumors (see Supporting Discussion (Text S1) for a detailed description).

As mentioned in the Methods section (see above), expression data from 12 patients were excluded from final analysis due to treatment differences. Thus, final sample size was 73 cases (146 paired pre- and post-chemotherapy samples). Nevertheless, we performed a parallel analysis including all patients recruited (85 cases, 170 paired pre- and post-chemotherapy samples). Highly significant correlations between both datasets (P < 0.001) (data not shown) highlighted the statistical robustness of the data.

**Figure 5:**
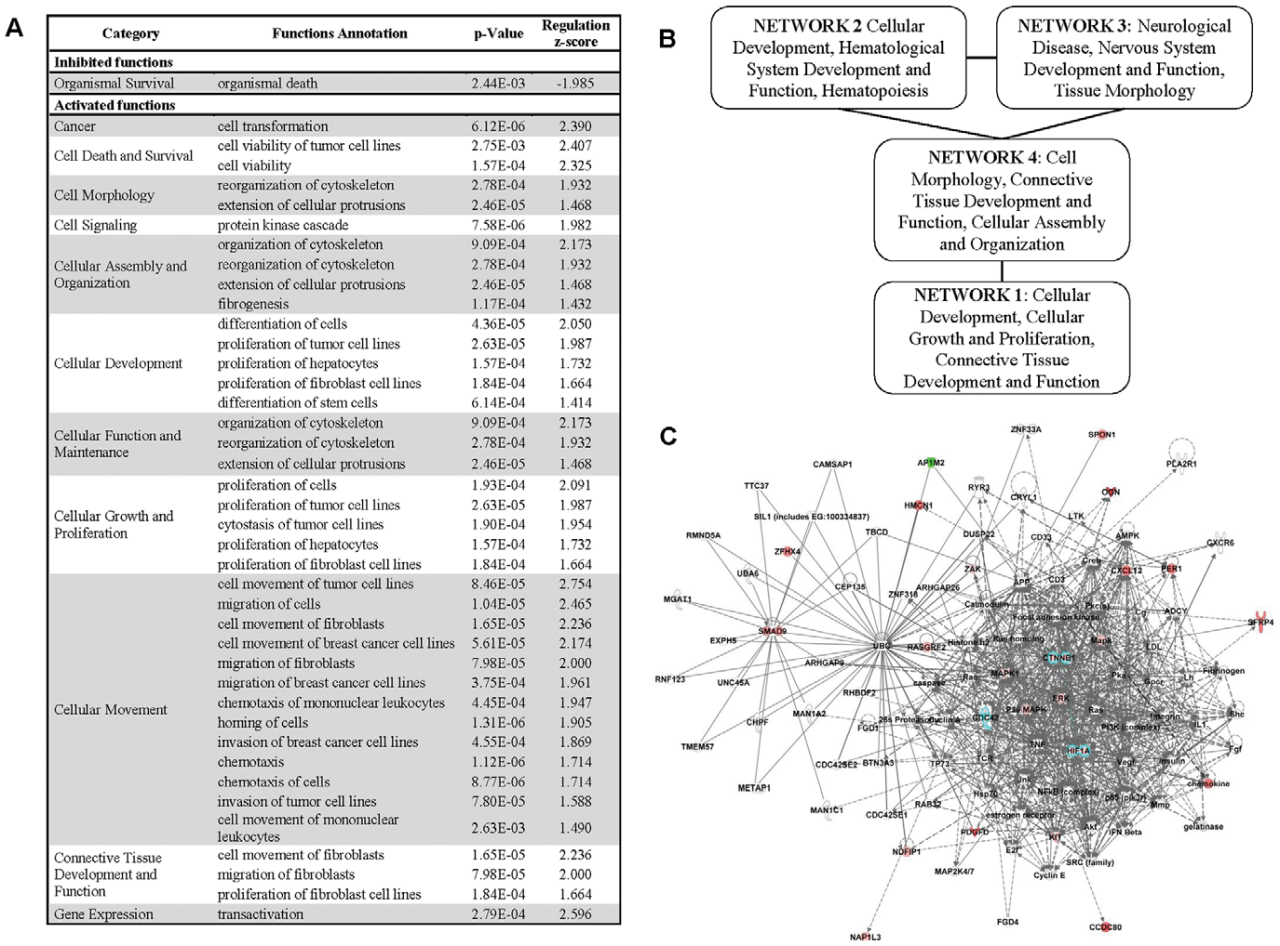
Functional annotation and network analysis of the Chemoresitance dataset by IPA software. A) List of predicted inhibited and activated functions according the Chemoresistance dataset. B) Summary of the IPA network analysis of the Chemoresistance dataset C) Gene-expression network resulting from merging overlapping Networks 2, 3 and 4 according to IPA network analysis.

**Figure 6:**
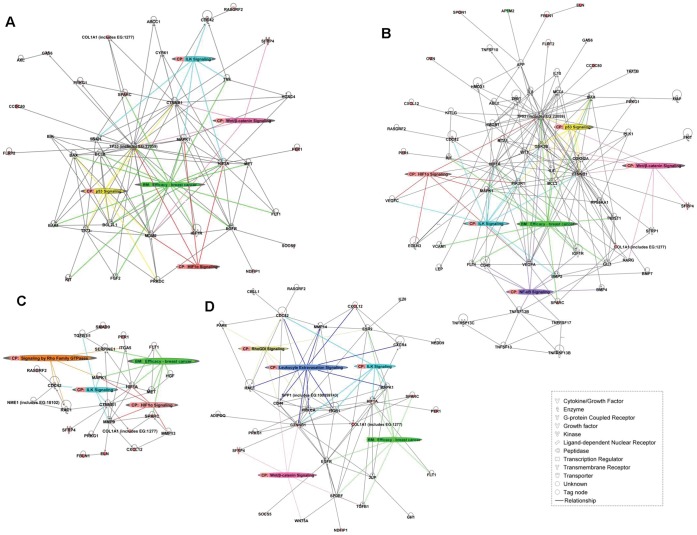
Gene-expression regulatory networks of breast cancer chemoresistance. Pathway analysis by IPA software based on the Chemoresistance dataset and gene lists related to A) Chemoresistance, B) Survival, C) ECM invasion and remodeling, and D) Migration created using the Ingenuity Knowledge Base. The relations between the genes were inferred from the relationships known in the scientific literature using data-mining Ingenuity software. Each node represents a gene; red color denotes over-expressed genes; green color denotes down-expressed genes. The colors intensity appears according to the related expression level by fold change. Connections indicate direct regulatory interactions. Arrows are colored differently to ease the identification of the genes involved in over-represented Canonical Pathways and Biomarkers according to Ingenuity Knowledge Base.

**Figure 7:**
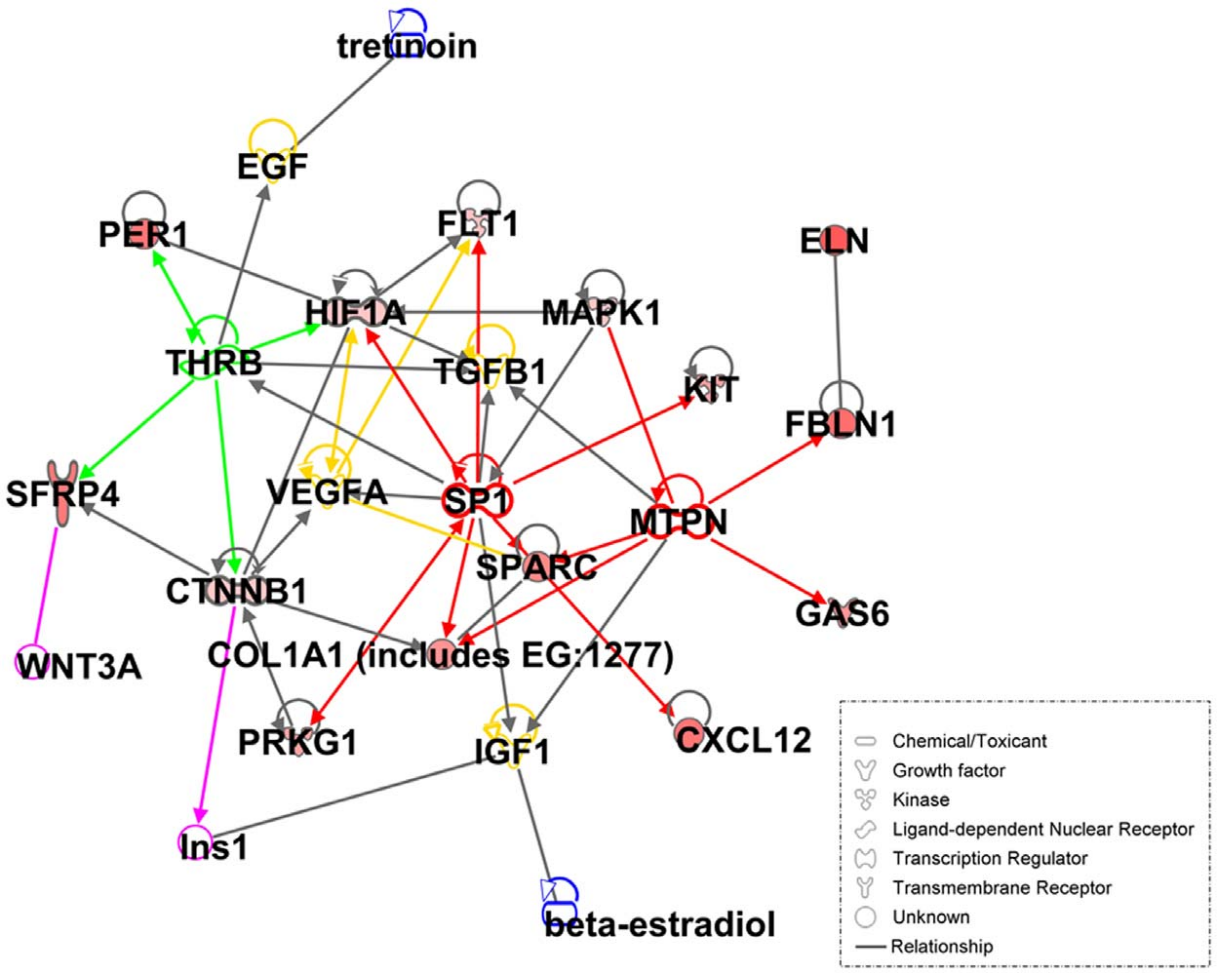
Upstream regulators analysis by IPA software based on the Chemoresistance dataset. Each ode represents a gene; red color denotes over-expressed genes. The colors intensity appears according to the related expression level by fold change. Connections indicate direct regulatory interactions. Arrows are colored differently to ease the identification of each connection.

Finally, IPA analysis allowed further insights into the molecular processes and pathways involved in breast cancer chemoresistance. According to IPA, the 30-gene set was enriched by genes related to cellular movement and migration, cell survival and connective tissue development and function involving tumor cells and fibroblasts. Additional important biological processes that were enriched in our gene set were related to growth and proliferation of tumor cells and fibroblasts, hematological system development and function (mostly regarding chemotaxis and blood cells aggregation) and cell morphology in relation to reorganization of the cytoskeleton. Consistent with these results, the only function close to significance which showed to be repressed is related to organismal death (Fig. 5A). IPA network analysis generated 4 networks related to previously described functions, further confirming the involvement of these processes in breast cancer chemoresistance. Networks 2, 3 and 4 were interconnected, whereas Network 1 was only related to Network 4 (Fig. 5B). Merging overlapping networks for pathways identification resulted in complicated models of direct and indirect interactions, so we focused on molecular pathways associated to the highlighted processes (see Supporting Methods (Text S1) for details). Interestingly, despite the complexity of the network resulting from the IPA analysis, we were able to observe that catenin (cadherin-associated protein), beta 1 (CTNNB1), hypoxia inducible factor 1 (HIF1) and CDC42 cell division cycle 42 (CDC42) occupied central positions in the network resulting from merging functionally related networks according to IPA analysis (Fig. 5C). Indeed, after analyzing the molecular relationships between our 30 target genes and genes known to be related with chemoresistance, cell survival, extracellular matrix invasion and remodeling and cellular migration we were able to observe that CTNNB1 and HIF1 continued occupying a central position within each network, whereas the rest of target genes tended to cluster around. Commonest canonical pathways indentified from this analysis were the Wnt/β-catenin signaling pathway and the HIF1 signaling pathway, together with p53 signaling pathways, Rho GTPases signaling pathways and some other pathways related to cytoskeleton and tissue remodeling. Worthy of note is the presenceidentification of efficacy biomarkers for breast cancer treatment among the genes participating in each gene expression network (Fig. 6). Lastly, IPA analysis suggested that the expression of the 30-gene set related to chemoresistance in our experimental system may be promoted by 4 growth factors - Transforming growth factor beta 1 (TGF-β1), Insulin Growth Factor 1 (IGF1), Vascular Endothelial Growth Factor A (VEGFA) and Epithelial Growth Factor (EGF)- , 2 transcriptional factors – Myotrophin (MTPN) and Sp1 transcription factor (Sp1)- and 1 ligand-dependent nuclear receptor - Thyroid Hormone Receptor, Beta (THRB)- (Fig. 7).

## Discussion

During the last decade, many efforts have focused on the identification of a gene signature able to either predict patient’s prognosis or response to systemic therapies against breast cancer. The main objective of such studies was to identify those patients who would clearly receive a benefit from cytotoxic therapies from those who could safely avoid this treatment [2], [23]. That work came to fruition in several commercial prognostic multigene classifiers, whose clinical utility is being assessed in large prospective clinical trials [24], [25]. Despite initial enthusiasm, it has been shown that the prognostic abilities of microarrays-derived gene signatures are complementary to traditional clinicopathological markers in clinical practice and treatment decision-making [26]. Multigene predictors for response to chemotherapy have been less successful, with no one of them commercially available or being tested for clinical utility [23]. Most of these studies analyzed tumor samples taken before chemotherapy and correlated the resulting gene expression data with the rate of pathological complete response (it would correspond to Miller & Payne grade 5 exclusively). Therefore the scientific novelty added by this work is that, from the best of our knowledge, this is the first time that a defined set of genes are reported to be expressed across breast tumors that show different histopathological responses to chemotherapy. Moreover, the main objective of the present study was not the prediction of response to chemotherapy, instead, we were mainly interested in the discovery of genetic markers for chemoresistance. This prompted us to include paired pre-/post-chemotherapy samples in order to observe significant gene expression changes selected by chemoresistant cancer cells to cope with cytotoxicity, which is a very different approach of that followed by preceding predictive genomic studies. Notably, this assay was not biased for individual genetic differences, as most samples used corresponded to paired pre- and post-treatment samples from each case.

We have shown that a set of 30 genes, functionally related with extracellular matrix metabolism, angiogenesis and developmental processes, undergo a positive switch in response to anthracycline and taxane-based chemotherapy in those patients who achieved a good pathological response to the treatment. The magnitude of the change reached up to a point to which transcriptional levels became similar to those found in patients that registered a poor histopathological response to the same treatment. These data suggest that those cells able to overcome chemotherapy and survive select the activation of a common transcriptional program, regardless primary response to chemotherapy. Then, the activation of this transcriptional program may draw the underlying molecular scenario that enable disease relapse despite chemotherapy-derived tumor shrinkage, in both good and bad responders, and reflect driving genetic and epigenetic aberrations which render chemoresistance. As pointed out previously [27], breast tumors are often composed of a mosaic of transformed cells among which only a rare subclone may overcome chemotoxicity. In the present study we target for analysis the cells within the residual tumor tissue after chemotherapy, which may correspond to those cells harboring selected genetic aberrations to resist the cytotoxic effect of chemotherapy.

Regarding biological function, the genes selected for the validation assay were involved in key biological processes for breast cancer invasion, among which, extracellular matrix metabolism, angiogenesis and developmental processes worth to be highlighted. Recently, several works has showed the relevance of tumor-microenvironment interactions in tumor chemoresistance and clinical outcome in breast cancer [28]-[30]. With respect to angiogenesis, it is well-known the involvement of this process into breast cancer invasion and disease progression [31]. On the other hand, some of the genes classified in the developmental processes category have been shown to participate in molecular pathways described in cancer stem cells (CSCs) [32]-[35]. Further functional characterization was performed using IPA software. Pathways analysis revealed that transcriptome networks, including our target genes and genes related to chemoresistance, cell survival and migration, and extracellular matrix remodeling and invasion, clustered around two central genes: CTNNB1 and HIF1. β-catenin, the protein encoded by CTNNB1, plays a key role in the regulation of mammary development through a dual role. In plasma membrane, β-catenin associates to cadherins forming cell-cell adherens junctions, which maintain mammary epithelial integrity. Loss of β-catenin from adherens junctions results in its elevation in cytosol and nucleus, where it regulates the expression of genes involved in mammary stem cell biology and breast development. Importantly, both of these events have been related to breast carcinogenesis and progression [36], [37]. The nuclear activity of β-catenin is promoted by several effectors through different signaling pathways. Transforming Growth Factor β (TGFβ) and several Wnt proteins are known to reduce β-catenin localization to adherens junctions by increasing the expression of transcription factors from the ZEB, SNAIL, and TWIST protein families and inducing the epithelial-to-mesenchymal transition (EMT) program. The EMT is a latent embryonic process converting epithelial cells into mesenchymal cells with enhanced motility and invasiveness capabilities [38]. Physiologically, EMT is related to organogenesis but experimental evidences have shown that the EMT process is linked to breast carcinogenesis and progression since it promotes the acquisition of stem-like properties, chemoresistance and metastasis [38], [39]. In addition, β-catenin localization to the nucleus is promoted by the kinase activity of growth factors receptors, such as those of the Epidermal Growth Factor (EGF) or the Insulin-like Growth Factor (IGF) families, components of the Nuclear Factor kappa B (NFκB) signaling pathway and the integrin-linked kinase (ILK) signaling pathway, which inhibits β-catenin targeting to proteasomal destruction [40]. Interestingly, both the ILK and the NFκB signaling pathways have been related with the Wnt/ β-catenin signaling pathway in our pathways analysis. Finally, increased tumor motility conferred by EMT requires changes in cell shape and polarity, which are mainly mediated by Rho Family GTPases activation via Wnt/ β-catenin signaling [41], as reflected in our pathways analysis. These facts, together with the identification of TGFβ1, IGF1 and EGF as potential upstream regulators of our 30 target genes, highlights the relevance of the molecular network centered on β-catenin for chemoresitance in breast cancer and its connections with the EMT process and CSCs biology, a hypothesis that warrants further investigation.

On the other hand, the HIF family comprises several transcription factors involved in the cellular adaptation to hypoxia through the modulation of key processes in tumor initiation and progression, such as angiogenesis, cell survival, metabolic reprogramming and therapeutic resistance. Adaptation to hypoxic conditions is crucial for tumor development and disease progression, since oxygen deprivation is a common microenvironmental feature of solid tumors and it increases as tumor growths [42]. Indeed, tumor hypoxia and consequent HIF-1 overexpression have been significantly correlated with worse clinical outcomes in cancer patients [43]. The molecular bases of these clinical observations rely on a complex network of interactions affecting HIF-1 transcription upon physicochemical stimuli, as low oxygen tension, increased reactive oxygen species (ROS) concentrations, growth factor and cytokine signaling [42]. Recent works support the idea that cancer chemoresistance is mediated by HIF-1-driven inactivation of intact p53 [44], [45] and activation of NFκB [45]. In line with these observations, our pathways analysis shows a connection between p53, NFκB and HIF-1 signaling pathways in relation to chemoresistance and survival. Regarding tumor metabolism under hypoxic conditions, it has recently been found that Sp1 cooperates with HIF-1 to promote glycolysis in solid tumors and thus, facilitating cancer metabolic reprogramming and tumor progression [46]. Similarly, Thyroid hormone has been shown to induce HIF-1α expression through THRB/Retinoid X receptor α (RXRα)-dependent activation of the Hepatic leukemia factor (HLF) in human hepatocytes and Hep2 cells [47], a possible role in breast cancer metabolism according to the results of our upstream regulator analysis with IPA software. In relation to MTPN, a protein related to cardiac hypertrophy, no significant relation with cancer has been reported to our knowledge. Additionally, some recent works point out the role of HIF-1 in mediating EMT through activation of the Wnt/β-catening signaling pathway. In 2008, Cannito et al. [48] reported that exposure cancer cells of different epithelial origin (hepatoma, pancreas, colon and breast carcinoma) to hypoxia invariably resulted in EMT. Based on their *in vitro* experiments, the authors proposed a mechanistic model by which early EMT events were induced by an increase in intracellular ROS production due to hypoxia, whereas late migration and invasiveness traits were promoted by HIF-1 through VEGF overexpression. Later studies have corroborated a significant enrichment in cell populations exhibiting stem like and EMT phenotypes after exposure to hypoxia [49], [50] and very recently, Conley et al [51] have proposed that tumor hypoxia secondary to antiangiogenic therapy in breast cancer limits its effectiveness, as it stimulates stem-like cell enrichment through HIF1-driven EMT.

These new data and concepts are integrated into the current debate regarding CSCs origin and biology. The hierarchical CSCs model proposed that oncogenesis is initiated by the occurrence of transforming mutations in normal stem cells (SC), which are transferred to their progeny following a hierarchical and unidirectional path. As a consequence, most progenitors and differentiated tumor cells are generated by self-renewal and differentiation of CSCs. Conceptually, this model presents some limitations since the mutation rate of SC might not be sufficiently high to promote oncogenesis. Main reasons are the small size of SC population and their quiescent proliferative state. These considerations and the biological insights coming from the discovery and study of the EMT process in cancer have pointed out cancer phenotypic plasticity as a major force directing oncogenesis and tumor progression. The dedifferentiation capacity of cancer cells to a stem-like phenotype through EMT conciliates the CSC and the multi-step tumorigenesis models, since mutations are more likely to strike actively dividing cells, which are later selected for clonal expansion or introduced into the CSCs compartment via EMT and upon the appropriate stimuli. Interestingly, microenvironmental factors, such as hypoxia, seem to trigger EMT in cancer and promote chemoresistance [39], [51].

Future studies will tell whether this set of genes for chemoresistance is constitutively expressed by a minority of aggressive cells within chemo-sensitive tumors or it is up-regulated in response to treatment-derived cytotoxicity. Similarly, further characterization of the cells expressing this set of genes will reveal their stemness. Indeed, they seem to share important features with undifferentiated cells since they are chemoresistant, represent a minor cell population within breast tumors, and show the up-regulation of genes functionally related with micro-environmental signaling pathways involved in the acquisition of motility and invasiveness traits [34], [35]. In any case, these data suggest that chemotherapy elicits a selective pressure able to activate the expression of adaptive capabilities in a selected population within the tumors whose proportion may vary depending on its molecular characteristics.

Then, the genes reported to differentially over-express after chemotherapy are ideal candidates for functional analysis to test their suitability as therapeutic targets itself or through the disruption of the molecular pathway in which they participate. Additionally, the study of molecular mechanism leading to the overexpression of these genes may shed light on the genetic aberrations leading chemoresistance. On the other hand, the development of a predictor based on the expression levels of these genes, or a selection of them, may support treatment decision-making in breast cancer, as it would help to identify those patients bearing chemosensitive tumors from those whose absolute benefit from current chemotherapy is very scarce, but still suffer from the severe toxicity associated to the use of cytostatics and other chemotherapeutic drugs. This strategy may allow a better selection of candidate breast cancer patients to be included in clinical trials testing new drugs.

### Accession Numbers

Microarray data have been submitted to the Gene Expression Omnibus (GEO) under accession number GSE28844.

## Supporting Information

Figure S1 **Summary of the methodology and main findings.**
(TIF)

Table S1 Differentially over-expressed genes after chemotherapy taking all pathological response groups.
(DOCX)

Table S2 Genes of interest selected for validation assay.
(DOCX)

Table S3 Genes differentially expressed after chemotherapy within each experimental group. RQ describes the magnitude of change of each target gene after chemotherapy with respect its expression before chemotherapy.
(DOCX)

Table S4 Genes differentially over-expressed after chemotherapy within the GR group –GR (Post-QT vs Pre-QT) comparison-.
(DOCX)

Table S5 Genes differentially expressed before chemotherapy between the GR and BR groups –Pre-QT (GR vs BR) comparison-.
(DOCX)

Table S6 Genes differentially expressed between experimental groups before chemotherapy. RQ describes the magnitude of change of each target gene with respect its expression in the experimental group corresponding to the second term of the comparison.
(DOCX)

Table S7 Genes differentially expressed between experimental groups after chemotherapy.
(DOCX)

Text S1 Supporting text containing Supporting Methods and Supporting Discussion.
(DOC)
