# Supplementary figures and images for: Transcriptional Shift Identifies a Set of Genes Driving Breast Cancer Chemoresistance
Source: PLoS One. 2013 Jan 10;8(1):e53983. doi: 10.1371/journal.pone.0053983 (PMC3542325; doi:10.1371/journal.pone.0053983)

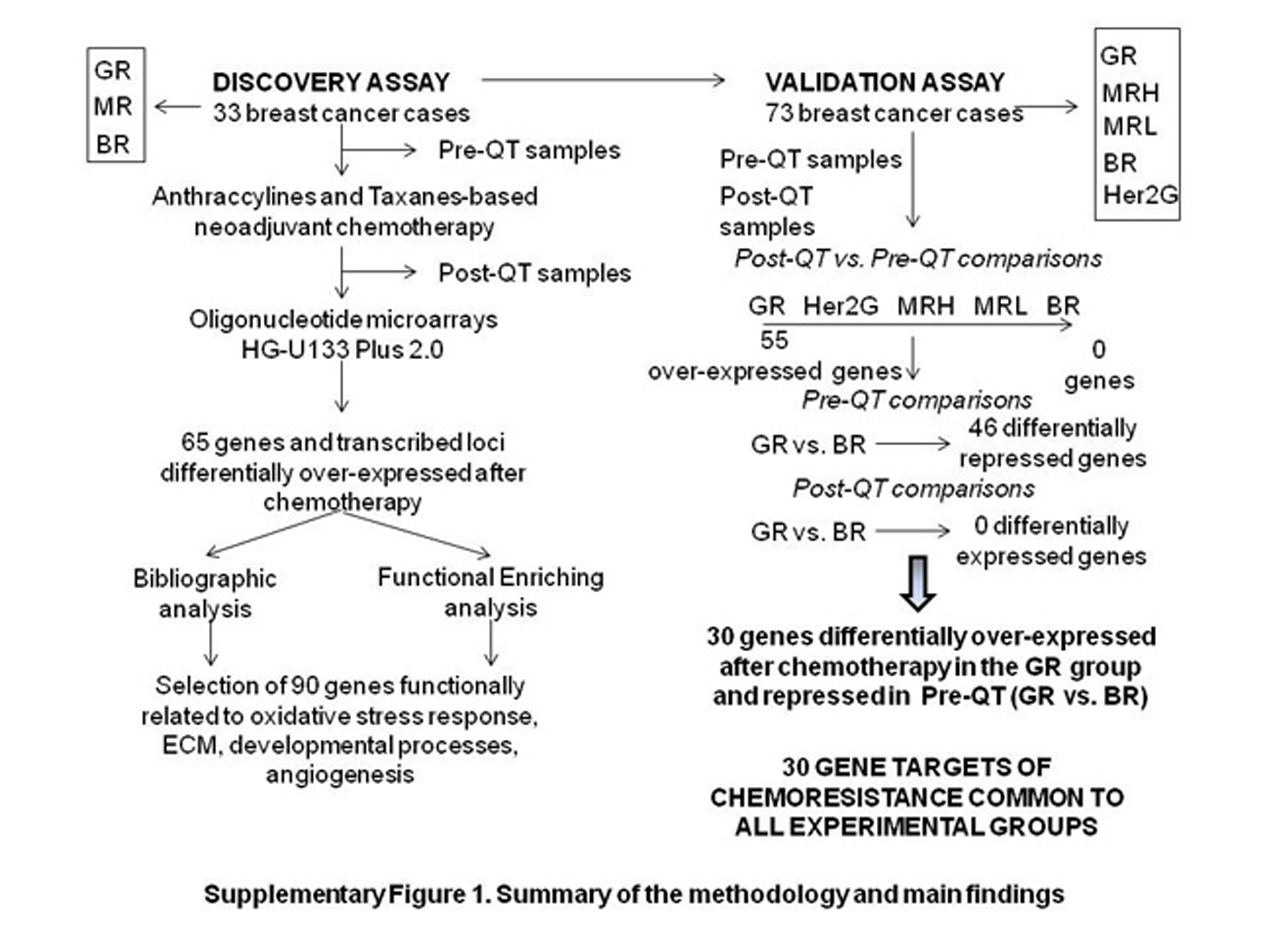

Supplement: Figure S1 — Summary of the methodology and main findings. (TIF) [file pone.0053983.s001.tif]
