# Supplementary material for: Transcriptional Shift Identifies a Set of Genes Driving Breast Cancer Chemoresistance
Source: PLoS One. 2013 Jan 10;8(1):e53983. doi: 10.1371/journal.pone.0053983 (PMC3542325; doi:10.1371/journal.pone.0053983)
Supplement: Table S1 — Differentially over-expressed genes after chemotherapy taking all pathological response groups. (DOCX) [file pone.0053983.s002.docx]

**Table S1.** Differentially over-expressed genes after chemotherapy taking all pathological response groups.

| **AFFY ID** | **Gene Symbol (UniGene)** | **P-value (Bonferroni)** | **P-value (FDR)** | **FC** |
| --- | --- | --- | --- | --- |
| 209189_at | FOS | 0.0202462 | 0.00040492 | 6.57593 |
| 228750_at | Transcribed locus | 0.00024893 | 0.00012446 | 5.31614 |
| 210764_s_at | CYR61 | 0.00238832 | 0.00012814 | 4.65884 |
| 222722_at | OGN | 0.00306971 | 0.00012814 | 4.42838 |
| 218730_s_at | OGN | 0.00145993 | 0.00012808 | 4.34352 |
| 227404_s_at | EGR1 | 0.00868799 | 0.00023481 | 3.89088 |
| 204052_s_at | SFRP4 | 0.00277324 | 0.00012814 | 3.79333 |
| 204051_s_at | SFRP4 | 0.00152836 | 0.00012808 | 3.75937 |
| 201041_s_at | DUSP1 | 0.00192122 | 0.00012808 | 3.72945 |
| 204359_at | FLRT2 | 0.00190595 | 0.00012808 | 3.69542 |
| 201289_at | CYR61 | 0.00439729 | 0.00015705 | 3.69293 |
| 202995_s_at | FBLN1 | 0.00266352 | 0.00012814 | 3.63798 |
| 212865_s_at | COL14A1 | 0.00999944 | 0.00023808 | 3.57203 |
| 227061_at | CDNA clone UTERU2015653 | 0.0338457 | 0.00057366 | 3.35518 |
| 214321_at | NOV | 0.00144675 | 0.00012808 | 3.33679 |
| 201694_s_at | EGR1 | 0.00142338 | 0.00012808 | 3.32085 |
| 202994_s_at | FBLN1 | 0.00159841 | 0.00012808 | 3.07057 |
| 243864_at | CCDC80 | 0.0174462 | 0.0003712 | 2.9816 |
| 1552289_a_at | CILP2 | 0.00264886 | 0.00012814 | 2.97854 |
| 212670_at | ELN | 0.00469104 | 0.00016176 | 2.95438 |
| 213993_at | SPON1 | 0.00301975 | 0.00012814 | 2.85407 |
| 213994_s_at | SPON1 | 0.0065725 | 0.00020539 | 2.83318 |
| 227209_at | CNTN1 | 0.0002148 | 0.00012446 | 2.73998 |
| 204472_at | GEM | 0.00865131 | 0.00023481 | 2.68147 |
| 204749_at | NAP1L3 | 0.00106731 | 0.00012808 | 2.5979 |
| 204457_s_at | GAS1 | 0.00307532 | 0.00012814 | 2.49752 |
| 219304_s_at | PDGFD | 0.00118699 | 0.00012808 | 2.47087 |
| 209436_at | SPON1 | 0.0128952 | 0.00029989 | 2.46483 |
| 236361_at | GALNT13 | 0.00364759 | 0.0001459 | 2.45128 |
| 205226_at | PDGFRL | 0.00085271 | 0.00012808 | 2.44452 |
| 227719_at | SMAD9 | 0.0164003 | 0.00035653 | 2.42699 |
| 201787_at | FBLN1 | 0.0150154 | 0.00033368 | 2.40659 |
| 222288_at | Transcribed locus | 0.0416578 | 0.00064898 | 2.39995 |
| 228640_at | PCDH7 | 0.0345477 | 0.00057579 | 2.35056 |
| 235944_at | HMCN1 | 0.0309136 | 0.00054234 | 2.32914 |
| 219779_at | ZFHX4 | 0.00137216 | 0.00012808 | 2.28599 |
| 217430_x_at | COL1A1 | 0.0279861 | 0.00051826 | 2.18803 |
| 223235_s_at | SMOC2 | 0.0185218 | 0.00038587 | 2.18388 |
| 1554007_at | CDNA clone IMAGE:5303689 | 0.00505853 | 0.00016862 | 2.16721 |
| 229331_at | SPATA18 | 0.0300625 | 0.00054234 | 2.1614 |
| 225871_at | STEAP2 | 0.00678533 | 0.00020562 | 2.13988 |
| 228109_at | RASGRF2 | 0.00960666 | 0.000235 | 2.06216 |
| *Table S1 continued* | | | | |
| 205880_at | PKD1 | 0.00701634 | 0.00020636 | 2.02661 |
| 219628_at | ZMAT3 | 0.00403645 | 0.0001495 | 1.96759 |
| 202177_at | GAS6 | 0.00391723 | 0.0001495 | 1.95287 |
| 226522_at | PODN | 0.0137785 | 0.00031315 | 1.93665 |
| 212667_at | SPARC | 0.0210836 | 0.00041341 | 1.93369 |
| 226932_at | SSPN | 0.0244706 | 0.00046171 | 1.93173 |
| 225725_at | CDNA clone IMAGE:5261213 | 0.00725772 | 0.00020736 | 1.87481 |
| 1598_g_at | GAS6 | 0.00244952 | 0.00012814 | 1.77547 |
| 228396_at | PRKG1 | 0.00963514 | 0.000235 | 1.76529 |
| 227221_at | CDNA clone IMAGE:5261213 | 0.00922199 | 0.000235 | 1.71563 |
| 204797_s_at | EMAP | 0.00208889 | 0.00012814 | 1.65215 |
| 227620_at | SLC44A1 | 0.00938248 | 0.000235 | 1.64053 |
| 209648_x_at | SOCS5 | 0.024051 | 0.00046171 | 1.61098 |
| 208127_s_at | SOCS5 | 0.0317073 | 0.00054668 | 1.60624 |
| 210102_at | VWA5A | 0.00189508 | 0.00012808 | 1.57861 |
| 222423_at | NDFIP1 | 0.0304418 | 0.00054234 | 1.48207 |
| 203794_at | CDC42BPA | 0.0198818 | 0.00040492 | 1.45802 |
| 230311_s_at | PRDM6 | 0.0408996 | 0.00064898 | 1.44695 |
| 203884_s_at | RAB11FIP2 | 0.00524894 | 0.00016932 | 1.40706 |
| 204020_at | PURA | 0.0384826 | 0.00062069 | 1.40121 |
| 226538_at | MAN2A1 | 0.00152848 | 0.00012808 | 1.37054 |
| 204021_s_at | PURA | 0.0421837 | 0.00064898 | 1.34598 |
| 205105_at | MAN2A1 | 0.0383403 | 0.00062069 | 1.32745 |

FC, fold change; FDR, false discovery rate.
