# Supplementary material for: Transcriptional Shift Identifies a Set of Genes Driving Breast Cancer Chemoresistance
Source: PLoS One. 2013 Jan 10;8(1):e53983. doi: 10.1371/journal.pone.0053983 (PMC3542325; doi:10.1371/journal.pone.0053983)
Supplement: Table S2 — Genes of interest selected for validation assay. (DOCX) [file pone.0053983.s003.docx]

**Table S2.** Genes of interest selected for validation assay.

| **Gene name** | **Gene Symbol** |
| --- | --- |
| **Genes selected on the basis of the results from the discovery assay** | |
| CDC42 binding protein kinase alpha (DMPK-like) | CDC42BPA |
| coiled-coil domain containing 80 | CCDC80 |
| collagen. type I. alpha 1 | COL1A1 |
| collagen. type XIV. alpha 1 (undulin) | COL14A1 |
| Contactin 1 | CNTN1 |
| cysteine-rich. angiogenic inducer. 61 | CYR61 |
| dual specificity phosphatase 1 | DUSP1 |
| Early growth response 1 | EGR1 |
| echinoderm microtubule associated protein like 1 | EML1 |
| elastin (supravalvular aortic stenosis. Williams-Beuren syndrome) | ELN |
| fibronectin leucine rich transmembrane protein 2 | FLRT2 |
| fibulin 1 | FBLN1 |
| growth arrest-specific 1 | GAS1 |
| growth arrest-specific 6 | GAS6 |
| GTP binding protein overexpressed in skeletal muscle | GEM |
| hemicentin 1 | HMCN1 |
| Nedd4 family interacting protein 1 | NDFIP1 |
| nephroblastoma overexpressed gene | NOV |
| nucleosome assembly protein 1-like 3 | NAP1L3 |
| osteoglycin | OGN |
| platelet derived growth factor D | PDGFD |
| platelet-derived growth factor receptor-like | PDGFRL |
| podocan | PODN |
| PR domain containing 6 | PRDM6 |
| protein kinase D1 | PRKD1 |
| protein kinase. cGMP-dependent. type I | PRKG1 |
| protocadherin 7 | PCDH7 |
| purine-rich element binding protein A | PURA |
| RAB11 family interacting protein 2 (class I) | RAB11FIP2 |
| Ras protein-specific guanine nucleotide-releasing factor 2 | RASGRF2 |
| secreted frizzled-related protein 4 | SFRP4 |
| secreted protein. acidic. cysteine-rich (osteonectin) | SPARC |
| six transmembrane epithelial antigen of the prostate 2 | STEAP2 |
| SMAD family member 9 | SMAD9 |
| SPARC related modular calcium binding 2 | SMOC2 |
| spondin 1. extracellular matrix protein | SPON1 |
| suppressor of cytokine signaling 5 | SOCS5 |
| Transcribed locus | SSPN |
| UDP-N-acetyl-alpha-D-galactosamine:polypeptide N-acetylgalactosaminyltransferase | GALNTL2 |
| *Table S2 continued* |  |
| v-fos FBJ murine osteosarcoma viral oncogene homolog | FOS |
| zinc finger homeobox 4 | ZFHX4 |
| **Genes selected following functional and bibliographic criteria** | |
| adaptor-related protein complex 1. mu 2 subunit | AP1M2 |
| ATP-binding cassette. sub-family B (MDR/TAP). member 1 | ABCB1 |
| ATP-binding cassette. sub-family G (WHITE). member 2 | ABCG2 |
| catenin (cadherin-associated protein). beta 1. 88kDa | CTNNB1 |
| CDP-diacylglycerol synthase (phosphatidate cytidylyltransferase) 1 | CDS1 |
| cell division cycle 42 (GTP binding protein. 25kDa) | cdc42 |
| chemokine (C-X-C motif) ligand 12 | CXCL12 |
| chemokine (C-X-C motif) receptor 4 | CXCR4 |
| endoplasmic reticulum metallopeptidase 1 | ERMP1 |
| epithelial cell transforming sequence 2 oncogene | ECT2 |
| family with sequence similarity 107. member A | FAM107A |
| focal adhesion kinase 1 | FAK1 |
| four and a half LIM domains 1 | FHL1 |
| GLI family zinc finger 1 | GLI1 |
| hypoxia inducible factor 1. alpha subunit | HIF1A |
| integrin. beta 1 (fibronectin receptor. beta polypeptide. antigen CD29 includes MDF2. MSK12) | ITGB1 |
| integrin. beta 3 (platelet glycoprotein IIIa. antigen CD61) | ITGB3 |
| integrin. beta 4 | ITGB4 |
| JUN N-terminal kinase | JNK |
| LIM and senescent cell antigen-like domains 2 | LIMS2 |
| lipase. hormone-sensitive | LIPE |
| mal. T-cell differentiation protein 2 | MAL2 |
| mast/stem cell growth factor receptor | SCFR |
| matrix metallopeptidase 9 (gelatinase B. 92kDa gelatinase. 92kDa type IV collagenase) | MMP9 |
| mitogen-activated protein kinase 1 | ERK2 |
| neuropilin 1 | NRP1 |
| NOTCH1 | Notch1 |
| nuclear factor of kappa light polypeptide gene enhancer in B-cells 1 | NF-KB1 |
| nucleolar and spindle associated protein 1 | NUSAP1 |
| p38 mitogen activated protein kinase | p38 |
| period homolog 1 (Drosophila) | PER1 |
| ras-related C3 botulinum toxin substrate 1 (rho family. small GTP binding protein Rac1) | Rac1 |
| serine peptidase inhibitor. Kunitz type. 2 | SPINT2 |
| signal transducer and activator of transcription 3 | STAT3 |
| sonic hedgehog | SHH |
| spectrin. beta. non-erythrocytic 1 | SPTBN1 |
| sterile alpha motif and leucine zipper containing kinase AZK | ZAK |
| Survivin | Survivin |
| tenascin XB | TNXB |
| *Table S2 continued* |  |
| topoisomerase (DNA) II alpha 170kDa | TOP2A |
| ubiquitin-like. containing PHD and RING finger domains. 1 | UHRF1 |
| v-akt murine thymoma viral oncogene homolog 1 | AKT1 |
| vascular endothelial growth factor A | VEGF-A165 |
| vascular endothelial growth factor C | VEGF-C |
| Supporting Table 2continued |  |
| vascular endothelial growth factor receptor 1 | VEGFR1 |
| vascular endothelial growth factor receptor 2 | VEGFR2 |
| vascular endothelial growth factor receptor 3 | VEGFR3 |
| v-rel reticuloendotheliosis viral oncogene homolog A (avian) | REL A |
| ZW10 interactor | ZWINT |

Genes were selected according to the results of the discovery assay and bibliographic criteria focused on functional relations between significant over-expressed genes after chemotherapy
