# Supplementary material for: Transcriptional Shift Identifies a Set of Genes Driving Breast Cancer Chemoresistance
Source: PLoS One. 2013 Jan 10;8(1):e53983. doi: 10.1371/journal.pone.0053983 (PMC3542325; doi:10.1371/journal.pone.0053983)
Supplement: Table S3 — Genes differentially expressed after chemotherapy within each experimental group. RQ describes the magnitude of change of each target gene after chemotherapy with respect its expression before chemotherapy. (DOCX) [file pone.0053983.s004.docx]

**Table S3.** Genes differentially expressed after chemotherapy within each experimental group. RQ describes the magnitude of change of each target gene after chemotherapy with respect its expression before chemotherapy.

| **Gene** | **RQ _Post-CT vs Pre-CT_** | **RQ _GR (Post-CT vs Pre-CT)_** | **RQ _Her2G (Post-CT vs Pre-CT)_** | **RQ _MRH (Post-CT vs Pre-CT)_** | **RQ _MRL (Post-CT vs Pre-CT)_** | **RQ _BR (Post-CT vs Pre-CT)_** |
| --- | --- | --- | --- | --- | --- | --- |
| ABCB1 | 5.22051399 | 18.7863593 | - | - | - | - |
| AP1M2 | 0.27364557 | 0.18527341 | - | - | - | - |
| CCDC80 | 8.30583516 | 24.9035562 | 3.90303671 | 6.43526756 | - | - |
| CDC42 | - | 3.26993026 | - | - | - | - |
| CNTN1 | 4.20108014 | 9.21143666 | 7.45427943 | 6.14923525 | - | - |
| COL14A1 | 23.2869531 | 152.96929 | 18.8156475 | 14.9904183 | - | - |
| COL1A1 | 2.45216252 | 9.42556898 | - | - | - | - |
| CTNNB1 | - | 3.16379687 | - | - | - | - |
| CXCL12 | 4.96811391 | 17.7098848 | 6.17022871 | - | - | - |
| CXCR4 | 4.02204436 | 4.15573211 | - | - | - | - |
| CYR61 | 6.93700362 | 15.9942326 | - | 8.86887349 | - | - |
| DUSP1 | 39.4831454 | 118.634773 | 32.7759926 | 20.8912387 | - | - |
| EGR1 | 26.4279316 | 91.8892096 | 21.8833712 | 27.3501581 | - | - |
| ELN | 7.16091811 | 32.8078197 | - | 9.76823923 | - | - |
| EML1 | 2.03056068 | - | - | - | - | - |
| FAM107A | - | 43.7354836 | - | - | - | - |
| FBLN1 | 5.82187972 | 21.7898132 | 5.43612978 | - | - | - |
| FHL1 | 11.3890393 | 27.5995193 | 17.1902104 | - | - | - |
| FLRT2 | 7.30486874 | 8.25765462 | 15.8634541 | - | - | - |
| FLT1 | 2.93547717 | 3.66768961 | 4.11518979 | 4.40447562 | - | - |
| FOS | 87.349656 | 301.374689 | 41.0432755 | 82.0529634 | 314.027697 | - |
| GALNTL2 | 4.20992711 | 8.90195973 | - | - | - | - |
| GAS1 | 2.9599777 | 5.72289519 | 6.16970781 | 3.11734076 | - | - |
| GAS6 | 3.60424669 | 9.25756696 | - | 3.77475864 | - | - |
| *Table S3 continued* | | | | | |  |
| GEM | 6.4379977 | 20.1878305 | 3.54158778 | 9.43439628 | - | - |
| GLI1 | 7.30224584 | 12.6628601 | 22.4772188 | 8.2736397 | - | - |
| HIF1A | - | 3.23169083 | - | - | - | - |
| HMCN1 | 4.16308517 | 17.8855569 | - | 5.19892785 | - | - |
| ITGB4 | 2.36588586 | - | - | - | - | - |
| KDR | 1.91150111 | - | - | 3.48954532 | - | - |
| KIT | 4.38576218 | 7.21497578 | 5.79625781 | 4.37288902 | - | - |
| MAPK1 | 2.12582604 | 3.32065324 | - | - | - | - |
| MAPK8 | 1.78421539 | - | - | 3.432882 | - | - |
| NAP1L3 | 3.79074397 | 5.25859157 | - | - | - | - |
| NDFIP1 | - | 5.03897534 | - | - | - | - |
| NOTCH1 | 1.84473298 | - | - | - | - | - |
| NOV | 10.715444 | 14.7979853 | 7.16140454 | 6.91463005 | - | - |
| NRP1 | 3.27542538 | 4.94143776 | 3.3987206 | - | - | - |
| OGN | 17.1234556 | 74.4143783 | 24.3667438 | - | - | - |
| PDGFD | 5.8770087 | 35.7791181 | 15.5311265 | - | - | - |
| PDGFRL | 4.4314074 | 24.5159965 | - | - | - | - |
| PER1 | 4.6960959 | 18.8005316 | 7.19757026 | 3.04392075 | - | - |
| PODN | 8.72658555 | 26.4201888 | 11.9921999 | 10.2345441 | - | - |
| PRDM6 | 3.55948157 | - | 4.65997786 | - | - | - |
| PRKD1 | 3.17434281 | 7.18643302 | - | - | - | - |
| PRKG1 | 1.86172577 | 6.87755998 | - | - | - | - |
| RAB11FIP2 | 1.98973665 | - | - | - | - | - |
| RAC1 | - | 2.45128546 | - | - | - | - |
| RASGRF2 | 2.92452339 | 5.19824073 | - | - | - | - |
| *Table S3 continued* | | | | | |  |
| SFRP4 | 4.82783392 | 16.3994467 | 12.3260271 | 2.83353941 | - | - |
| SMAD9 | - | 9.43966808 | - | - | - | - |
| SOCS5 | 4.50483753 | 3.95622867 | 11.4290092 | - | - | - |
| SPARC | 3.50674038 | 12.0367405 | - | - | - | - |
| SPINT2 | - | - | 0.18345832 | - | - | - |
| SPON1 | 3.55313965 | 11.6892213 | - | - | - | - |
| SPTBN1 | 2.07215736 | 4.76568851 | - | - | - | - |
| SSPN | 4.55422422 | 11.4258243 | 7.73733586 | - | - | - |
| STEAP2 | 3.6660691 | 7.49707654 | 5.772502 | - | - | - |
| TNXB | 24.1926332 | 158.167929 | 19.9286356 | 10.0195522 | - | - |
| TOP2A | 0.11268083 | 0.04972546 | 0.0602591 | - | - | - |
| UHRF1 | 0.43312899 | - | - | - | - | - |
| VEGFA | 0.45549202 | - | - | - | - | - |
| VEGFC | 2.46132279 | 5.31736888 | - | - | - | - |
| ZAK | 1.83785984 | 3.30297386 | - | - | - | - |
| ZFHX4 | 4.81722371 | 11.9025459 | 7.96945002 | - | - | - |
| Total | 57 genes | 55 genes | 30 genes | 22 genes | 1 gen | 0 genes |

BR, bad response group; GR, good response group; Her2G, Her2-positive group; MRH, mid-response high group; MRL, mid-response low group; Post-CT, after chemotherapy; Pre-CT, before chemotherapy; RQ, relative quantity.
