# Supplementary material for: Transcriptional Shift Identifies a Set of Genes Driving Breast Cancer Chemoresistance
Source: PLoS One. 2013 Jan 10;8(1):e53983. doi: 10.1371/journal.pone.0053983 (PMC3542325; doi:10.1371/journal.pone.0053983)
Supplement: Table S4 — Genes differentially over-expressed after chemotherapy within the GR group –GR (Post-QT vs Pre-QT) comparison-. (DOCX) [file pone.0053983.s005.docx]

**Table S4.** Genes differentially over-expressed after chemotherapy within the GR group –GR (Post-CT vs Pre-CT) comparison-.

| **Gene** | **RQ _GR (Post-CT vs Pre-CT)_** | |
| --- | --- | --- |
| ABCB1 | 18.7863593 | |
| AP1M2 | 0.18527341 | |
| CCDC80 | 24.9035562 | |
| CDC42 | 3.26993026 | |
| CNTN1 | 9.21143666 | |
| COL14A1 | 152.96929 | |
| COL1A1 | 9.42556898 | |
| CTNNB1 | 3.16379687 | |
| CXCL12 | 17.7098848 | |
| CXCR4 | 4.15573211 | |
| CYR61 | 15.9942326 | |
| DUSP1 | 118.634773 | |
| EGR1 | 91.8892096 | |
| ELN | 32.8078197 | |
| FAM107A | 43.7354836 | |
| FBLN1 | 21.7898132 | |
| FHL1 | 27.5995193 | |
| FLRT2 | 8.25765462 | |
| FLT1 | 3.66768961 | |
| FOS | 301.374689 | |
| GALNTL2 | 8.90195973 | |
| GAS1 | 5.72289519 | |
| GAS6 | 9.25756696 | |
| GEM | 20.1878305 | |
| GLI1 | 12.6628601 | |
| HIF1A | 3.23169083 | |
| HMCN1 | 17.8855569 | |
| KIT | 7.21497578 | |
| MAPK1 | 3.32065324 | |
| NAP1L3 | 5.25859157 | |
| NDFIP1 | 5.03897534 | |
| NOV | 14.7979853 | |
| NRP1 | 4.94143776 | |
| OGN | 74.4143783 | |
| PDGFD | 35.7791181 | |
| PDGFRL | 24.5159965 | |
| PER1 | 18.8005316 | |
| PODN | 26.4201888 | |
| PRKD1 | 7.18643302 | |
| PRKG1 | 6.87755998 | |
| RAC1 | 2.45128546 | |
| RASGRF2 | 5.19824073 | |
| SFRP4 | 16.3994467 | |
| *Table S4 continued* | | |
| SMAD9 | 9.43966808 | |
| SOCS5 | 3.95622867 | |
| SPARC | 12.0367405 | |
| SPON1 | 11.6892213 | |
| SPTBN1 | 4.76568851 | |
| SSPN | 11.4258243 | |
| STEAP2 | 7.49707654 | |
| TNXB | 158.167929 | |
| TOP2A | 0.04972546 | |
| VEGFC | 5.31736888 | |
| ZAK | 3.30297386 | |
| ZFHX4 | 11.9025459 | |

Relative quantity (RQ) describes the magnitude of change of each target gene after chemotherapy with respect its expression before chemotherapy. GR, good response group; Post-CT, after chemotherapy; Pre-CT, before chemotherapy; RQ, relative quantity.
