# Supplementary material for: Transcriptional Shift Identifies a Set of Genes Driving Breast Cancer Chemoresistance
Source: PLoS One. 2013 Jan 10;8(1):e53983. doi: 10.1371/journal.pone.0053983 (PMC3542325; doi:10.1371/journal.pone.0053983)
Supplement: Table S5 — Genes differentially expressed before chemotherapy between the GR and BR groups –Pre-QT (GR vs BR) comparison-. (DOCX) [file pone.0053983.s006.docx]

**Table S5** Genes differentially expressed before chemotherapy between the GR and BR groups –Pre-QT (GR vs BR) comparison-.

| **Gene** | | **RQ _Pre-QT (GR vs BR)_** | |
| --- | --- | --- | --- |
| AP1M2 | | 0.255125214 | |
| BIRC5 | | 0.110677577 | |
| CCDC80 | | 0.220793548 | |
| CDC42 | | 0.206385939 | |
| CDS1 | | 0.082805419 | |
| COL1A1 | | 0.125509807 | |
| CTNNB1 | | 0.192678056 | |
| CXCL12 | | 0.187189206 | |
| ELN | | 0.050310318 | |
| ERMP1 | | 0.20689296 | |
| FBLN1 | | 0.120018468 | |
| FLRT2 | | 0.081622721 | |
| FLT1 | | 0.221115432 | |
| GAS6 | | 0.18383045 | |
| HIF1A | | 0.196784302 | |
| HMCN1 | | 0.17215669 | |
| ITGB1 | | 0.21352813 | |
| ITGB4 | | 0.167066634 | |
| KIT | | 0.187318823 | |
| MAL2 | | 0.282348031 | |
| MAPK1 | | 0.21790637 | |
| MAPK14 | | 0.249461483 | |
| MAPK8 | | 0.226563528 | |
| NAP1L3 | | 0.115303297 | |
| NDFIP1 | | 0.118071989 | |
| NFKB1 | | 0.243706413 | |
| NOTCH1 | | 0.215517053 | |
| OGN | | 0.076928492 | |
| PDGFD | | 0.048857703 | |
| PER1 | | 0.104646914 | |
| PRKG1 | | 0.100946291 | |
| PTK2 | | 0.139123609 | |
| PURA | | 0.171486164 | |
| RASGRF2 | | 0.172151561 | |
| REL | | 0.261837755 | |
| SFRP4 | | 0.166182257 | |
| SMAD9 | | 0.063640678 | |
| SOCS5 | | 0.212031403 | |
| SPARC | | 0.099342014 | |
| SPINT2 | | 0.254468059 | |
| SPON1 | | 0.075141141 | |
| SSPN | | 0.104315474 | |
| STAT3 | | 0.151972732 | |
| *Table S5 continued* | |  |  |
| VEGFA | | 0.213175336 | |
| ZAK | | 0.233274662 | |
| ZFHX4 | | 0.09150727 | |

Relative quantity (RQ) describes the magnitude of change of each target gene before chemotherapy in the GR group with respect the BR group. BR, bad response group; GR, good response group; Pre-QT, before chemotherapy; RQ, relative quantity.
