# Supplementary material for: Transcriptional Shift Identifies a Set of Genes Driving Breast Cancer Chemoresistance
Source: PLoS One. 2013 Jan 10;8(1):e53983. doi: 10.1371/journal.pone.0053983 (PMC3542325; doi:10.1371/journal.pone.0053983)
Supplement: Table S6 — Genes differentially expressed between experimental groups before chemotherapy. RQ describes the magnitude of change of each target gene with respect its expression in the experimental group corresponding to the second term of the comparison. (DOCX) [file pone.0053983.s007.docx]

**Table S6.** Genes differentially expressed between experimental groups before chemotherapy. RQ describes the magnitude of change of each target gene with respect its expression in the experimental group corresponding to the second term of the comparison.

| **Gene** | **RQ_GR vs MRH_** | **RQ_GR vs MRL_** | **RQ_GR vs BR_** | **RQ_Her2G vs GR_** | **RQ_Her2G vs MRH_** | **RQ_Her2G vs MRL_** | **RQ_Her2G vs BR_** | **RQ_MRH vs BR_** | **RQ_MRH vs MRL_** | **RQ_MRL vs BR_** |
| --- | --- | --- | --- | --- | --- | --- | --- | --- | --- | --- |
| ABCG2 | - | 0.0455904 | 0.255125214 | - | - | - | - | - | - | - |
| AP1M2 | - | 0.2011848 | 0.110677577 | - | - | - | - | - | - | - |
| BIRC5 | - | 0.0711531 | 0.220793548 | - | - | - | - | - | - | - |
| CCDC80 | - | 0.179527831 | 0.206385939 | - | - | - | - | - | - | - |
| CDC42 | - | 0.264425523 | - | - | - | - | - | - | - | - |
| CDC42BPA | - | 0.291359961 | 0.082805419 | - | - | - | - | - | - | - |
| CDS1 | - | - | - | - | - | - | - | - | - | - |
| CNTN1 | - | 0.173990342 | 0.125509807 | - | - | - | - | - | - | - |
| COL1A1 | - | 0.091596038 | 0.192678056 | - | - | - | - | - | - | - |
| CTNNB1 | - | 0.248095234 | 0.187189206 | - | - | - | - | - | - | - |
| CXCL12 | - | 0.233231044 | 0.050310318 | - | - | - | - | - | - | - |
| ELN | - | 0.109152522 | - | - | - | - | - | - | - | - |
| EML1 | - | 0.2366714 | 0.20689296 | - | - | - | - | - | - | - |
| ERMP1 | - | 0.174301237 | 0.120018468 | - | - | - | - | - | - | - |
| FBLN1 | - | 0.102108019 | - | - | - | - | - | - | - | - |
| FHL1 | - | 0.100892539 | 0.081622721 | - | - | - | - | - | - | - |
| FLRT2 | - | 0.103333667 | 0.221115432 | - | - | - | 0.048494295 | - | - | - |
| FLT1 | - | 0.172172001 | 0.18383045 | - | - | - | - | - | - | - |
| GALNT2 | - | - | - | - | - | - | - | - | - | - |
| GAS6 | - | 0.1822029 | - | - | - | - | - | - | - | - |
| GLI1 | - | 0.130749842 | 0.196784302 | - | - | - | - | - | - | - |
| HIF1A | - | 0.170323546 | 0.17215669 | - | - | - | - | - | - | - |
| HMCN1 | - | 0.133705216 | 0.21352813 | - | - | - | - | - | - | - |
| ITGB1 | - | 0.185414851 | 0.167066634 | - | - | - | - | - | - | - |
| ITGB4 | - | - | - | - | - | - | - | - | - | - |
| KDR | - | 0.211097928 | 0.187318823 | - | - | - | - | - | - | - |
| KIT | - | - | 0.282348031 | - | - | - | - | - | - | - |
| MAL2 | - | - | 0.21790637 | - | - | - | - | - | - | - |
| MAPK1 | - | 0.244283689 | 0.249461483 | - | - | - | - | - | - | - |
| MAPK14 | - | 0.186427658 | 0.226563528 | - | - | - | - | - | - | - |
| *Table S6 continued* | | | | | | | | | | |
| MAPK8 | - | 0.211924929 | - | - | - | - | - | - | - | - |
| MMP9 | - | 0.140669904 | 0.115303297 | - | - | - | - | - | - | - |
| NAP1L3 | - | 0.153685842 | 0.118071989 | - | - | - | - | - | - | - |
| NDFIP1 | - | 0.126531981 | 0.243706413 | - | - | - | - | - | - | - |
| NFKB1 | - | - | 0.215517053 | - | - | - | - | - | - | - |
| NOTCH1 | - | - | - | - | - | - | - | - | - | - |
| NRP1 | - | 0.250803031 | 0.076928492 | - | - | - | - | - | - | - |
| OGN | - | - | 0.048857703 | - | - | - | - | - | - | - |
| PDGFD | - | 0.114661371 | - | - | - | - | 0.054955242 | - | - | - |
| PDGFRL | - | 0.171356188 | 0.104646914 | - | - | - | - | - | - | - |
| PODN | - | 0.082401112 | - | - | - | - | - | - | - | - |
| PRDM6 | - | 0.186491312 | - | - | - | - | - | - | - | - |
| PRKD1 | - | 0.093087275 | 0.100946291 | - | - | - | - | - | - | - |
| PRKG1 | - | - | 0.139123609 | - | - | - | - | - | - | - |
| PTK2 | - | - | 0.171486164 | - | - | - | - | - | - | - |
| PURA | - | 0.12299748 | - | - | - | - | - | - | - | - |
| RAB11FIP2 | - | 0.218605231 | 0.172151561 | - | - | - | - | - | - | - |
| RASGRF2 | - | 0.118278192 | 0.261837755 | - | - | - | - | - | - | - |
| REL | - | 0.188733777 | 0.166182257 | - | - | - | - | - | - | - |
| SFRP4 | - | 0.113632579 | 0.063640678 | - | - | - | - | - | - | - |
| SMAD9 | - | - | 0.212031403 | - | - | - | - | - | - | - |
| SOCS5 | - | 0.202196735 | 0.099342014 | - | - | - | - | - | - | - |
| SPARC | - | 0.103744815 | 0.254468059 | - | - | - | - | - | - | - |
| SPINT2 | - | - | 0.075141141 | - | - | - | - | - | - | - |
| SPON1 | - | 0.084121968 | 0.104315474 | - | - | - | - | - | - | - |
| SSPN | - | 0.144382805 | 0.151972732 | - | - | - | - | - | - | - |
| STAT3 | - | - | - | - | - | - | - | - | - | - |
| STEAP2 | - | 0.14829528 | 0.213175336 | - | - | - | - | - | - | - |
| VEGFA | - | 0.199504049 | - | - | - | - | - | - | - | - |
| VEGFC | - | 0.125948858 | 0.233274662 | - | - | - | - | - | - | - |
| ZAK | - | 0.266141246 | 0.09150727 | - | - | - | - | - | - | - |
|  |  |  |  |  |  |  |  |  |  |  |
| *Table S6 continued* | | | | | | | | | | |
| ZFHX4 | - | 0.087975346 | 0.255125214 | - | - | - | - | - | - | - |
| Total | 0 genes | 49 genes | 46 genes | 4 genes | 0 genes | 0 genes | 2 genes | 0 genes | 0 genes | 0 genes |

BR, bad response group; GR, good response group; Her2G, Her2-positive group; MRH, mid-response high group; MRL, mid-response low group; Post-QT, after chemotherapy; Pre-QT, before chemotherapy; RQ, relative quantity.
