# Supplementary material for: Transcriptional Shift Identifies a Set of Genes Driving Breast Cancer Chemoresistance
Source: PLoS One. 2013 Jan 10;8(1):e53983. doi: 10.1371/journal.pone.0053983 (PMC3542325; doi:10.1371/journal.pone.0053983)
Supplement: Table S7 — Genes differentially expressed between experimental groups after chemotherapy. (DOCX) [file pone.0053983.s008.docx]

**Table S7.** Genes differentially expressed between experimental groups after chemotherapy**.**

| Gene | RQ_GR vs MRH_ | RQ_GR vs MRL_ | RQ_GR vs BR_ | RQ_Her2G vs GR_ | RQ_Her2G vs MRH_ | RQ_Her2G vs MRL_ | RQ_Her2G vs BR_ | RQ_MRH vs BR_ | RQ_MRH vs MRL_ | RQ_MRL vs BR_ |
| --- | --- | --- | --- | --- | --- | --- | --- | --- | --- | --- |
| AP1M2 | - | 0.061647912 | - | - | - | - | - | - | - | - |
| BIRC5 | - | 0.030327218 | - | - | - | - | - | - | 0.072183112 | - |
| CCDC80 | - |  | - | - | - | - | - | - | 0.091471127 | - |
| COL1A1 | - |  | - | - | - | - | - | - | 0.129925048 | - |
| CTNNB1 | - |  | - | - | - | - | - | - | 0.150214663 | - |
| FBLN1 | - |  | - | - | - | - | - | - | 0.051737873 | - |
| FLRT2 | - | 0.060531833 | - | - | - | - | - | - | 0.057536593 | - |
| ITGB1 | - |  | - | - | - | - | - | - | 0.093779575 | - |
| MAPK1 | - |  | - | - | - | - | - | - | 0.11496204 | - |
| MMP9 | - |  | - | - | - | - | - | - | 0.075171727 | - |
| PRDM6 | - | 0.055055743 | - | - | - | - | - | - | - | - |
| PURA | - |  | - | - | - | - | - | - | 0.095695941 | - |
| SOCS5 | - | 0.109764099 | - | - | - | - | - | - | 0.091465914 | - |
| SPARC | - |  | - | - | - | - | - | - | 0.104708185 | - |
| SPINT2 | - | 0.035157415 | - | - | - | 0.040451006 | - | - | 0.05473013 | - |
| SPON1 | - |  | - | - | - | - | - | - | 0.087146283 | - |
| STEAP2 | - |  | - | - | - | - | - | - | 0.120035222 | - |
| TOP2A | - | 0.061987627 | - | - | - | - | - | - | - | - |
| VEGFA | - |  | - | - | - | - | - | - | 0.142822842 | - |
| Total | 0 genes | 7 genes | 0 genes | 0 genes | 0 genes | 1 gen | 0 genes | 0 genes | 16 genes | 0 genes |

RQ describes the magnitude of change of each target gene with respect its expression in the experimental group corresponding to the second term of the comparison. BR, bad response group; GR, good response group; Her2G, Her2-positive group; MRH, mid-response high group; MRL, mid-response low group; Post-CT, after chemotherapy; Pre-CT, before chemotherapy; RQ, relative quantity.
