# Supplementary material for: Transcriptional Shift Identifies a Set of Genes Driving Breast Cancer Chemoresistance
Source: PLoS One. 2013 Jan 10;8(1):e53983. doi: 10.1371/journal.pone.0053983 (PMC3542325; doi:10.1371/journal.pone.0053983)
Supplement: Text S1 — Supporting text containing Supporting Methods and Supporting Discussion. (DOC) [file pone.0053983.s009.doc]

**TEXT S1**

**Supporting Methods**

**Tumor tissue selection**

Tumor samples from each patient included in this study were obtained before and after chemotherapy. Before RNA extraction protocol, frozen tumor samples were sectioned in a cryostat to evaluate their celularity. 3 µm sections were cut for conventional hematoxylin-eosin staining to determine the following parameters: percentage of infiltrating tumor cells, percentage of in situ carcinoma, percentage of necrosis, percentage of normal epithelial cells, percentage of fibrosis, percentage of inflammatory cells, percentage of total cells in the biopsy and percentage of tumoral cells. Only those cases with ≥ 50% infiltrating tumor cells and scarce inflammatory or necrotic tissue in their pre-chemotherapy tumor samples were selected for RNA extraction. Genome-wide expression analysis using oligonucleotide microarrays was performed on 33 breast cancer cases, which were selected irrespectively of their Her2 receptor status, unless it was registered as a phenotypic tumor characteristic (Table 1). Formalin fixed paraffin embedded (FFPE) samples for validation assay underwent the same microscopic examination to be selected. In addition, matching pairs of pre-chemotherapy and post-chemotherapy FFPE tumor samples were examined by a pathologist who assessed the pathologic response to primary chemotherapy by using Miller & Payne grading system: 19 Her2-negative breast cancer cases with Miller & Payne grade 4 or 5 included in the “good response group”(GR), 34 Her2-negative breast cancer cases with Miller & Payne grade 3 included in a group we termed the “mid-response group” (MR) and 15 Her2-negative breast cancer cases with Miller & Payne grade 1 or 2 included in a group we termed the “bad response group” (BR). In the case of MR we further subdivided this group into 2 subgroups according the percentage of tumor cells elimination after chemotherapy: 17 Her2-negative breast cancer cases with Miller & Payne grade 3 and a percentage of tumor cell elimination between 30-60% -MR low (MRL)-. and 17 Her2-negative breast cancer cases with Miller & Payne grade 3 and a percentage of tumor cell elimination between 61-90% -MR high (MRH)-. We also included 17 Her2-positive breast cancer cases forming the “Her2 group” (Her2G).

This distribution was not chosen randomly, in such a way that the number of cases in each group mimics the distribution of breast cancer cases with respect pathologic response found in the registry of the Department of Pathology of the Hospital Medico-Quirurgico de Jaen from 2007 to 2010 (data not shown). In this sense, BR represents the 22% of the Her2-negative sample size, MR represents the 50% of the Her2-negative sample size and GR represents the 28% of the Her2-negative sample size. The Her2-positive extra group corresponds to the 20% of total sample size. All tumor samples were selected by an experienced pathologist on the basis of their celularity and histological characteristics.

Nevertheless, for clinical reasons, the sample size of this experiment was modified for data analysis. 12 cases (3 from BR group, 5 from MRL group, 2 from MRH group and 2 from VH group) were excluded in the final data analysis. In this case, BR represents the 20.7% of the Her2-negative sample size, MR represents the 46.5% of the Her2-negative sample size and GR represents the 32.8% of the Her2-negative sample size. The Her2-positive extra group corresponds to the 20.5% of total sample size. Then, patient’s distribution did not undergo mayor changes with respect the initial design.

**Gene selection for validation assay**

Based on the microarray results, expression levels of 90 genes were evaluated using qPCR analysis. These genes were selected according to functional criteria. First, of the 47 genes and 6 transcribed loci differentially over-expressed after chemotherapy, 41 were selected for validation assay according to their functional characteristics. After a careful study of the signaling pathways and biological processes in which these genes are involved, 49 additional genetic targets were added to the validation gene panel. NCBI resources ([http://www.ncbi.nlm.nih.gov](http://www.ncbi.nlm.nih.gov/gene)), the results from functional enrichment analysis, on-line tools such as Oncomine ([www.oncomine.org](http://www.oncomine.org/)) and GeneCards ([www.genecards.org](http://www.genecards.org/)), and the available literature were used to identify known or putative gene function. This strategy endowed the validation assay with a deeper functional design in order to preliminary identify the molecular cascades specifically deregulated in tumor chemo-resistant cells.

**IPA analysis**

A dataset containing gene identifiers and the corresponding expression values was uploaded into the Ingenuity Pathways Analysis (IPA) application. These genes, called the focus genes, were overlaid onto a global molecular network developed from information contained in the Ingenuity Knowledge Base. Networks of the focus genes were algorithmically generated based on their connectivity and expression values. Significance of each network and function was assessed using the Fisher’s exact tests (*P* ≤ 0.05). Functional and transcriptional regulation was predicted by the Activation z-score. The z-score is based on relationships in the molecular pathways (networks), which represent experimentally observed gene expression or transcription events and function annotation data, as derived from the information compiled in the Ingenuity Knowledge Base. These relationships are associated with a direction of change that is either activating (z-score ≥ 2) or inhibiting (z-score ≤ -2).

For pathways analysis, the Search feature in IPA was used to create gene lists associated with molecular and cellular processes of interest in cancer. The lists were denominated as Chemoresistance, Survival, ECM invasion and remodeling, and Migration, in accordance with the biological processes in which they participate. The Chemoresistance list included genes searched as “Drug resistance of tumor cell lines”, “Drug resistance of cancer cells”, “Chemotherapy resistance of tumor cell lines” and “Drug resistance of breast cancer cell lines” within the Functions and Diseases Search Category “Cellular Response to Therapeutics”. The Survival list included genes searched as “Cell viability of cancer cells” within the Functions and Diseases Search Category “Cell Death and Survival”. The ECM invasion and remodeling list included genes searched as “Invasion of extracellular matrix” and “Remodeling of extracellular matrix” within the Functions and Diseases Search Categories “Cancer” and “Tissue Morphology”, respectively. The Migration list included genes searched as “Migration of mammary tumor cells” within the Functions and Diseases Search Category “Cellular Movement”. Finally, we added our 30-gene set to each list and constructed a graphical model using the Pathways building tools within the My Pathways feature in IPA. The Connect tool was used to gather genes into a module of molecules tightly connected by direct protein interactions and regulatory events, according to the Ingenuity knowledge base, and the Overlay tool was employed to integrate the expression profile of our data set, together with adjacent signaling/metabolic pathways and the identification of some of the genes as potential biomarkers in the clinics. As a result, we obtained a graphical representation of the main molecular interactions and pathways potentially regulating chemoresistance in our study population.

Transcriptional regulation analysis was performed using the IPA Upstream regulators tool. Upstream regulators showing a significant *P*-value and a z-score not lower than 1.4, were exported to My Pathways feature. As previously, we added our 30-gene set and explored the potential direct functional connections among selected genes, using the Pathways building tools. We decided to extend our selection to those regulators not strictly predicted to be activated by z-score algorithm but near to significance, given the chance of losing significant information due the small size of our target gene set.

**Supporting Discussion**

**Intermediate-response groups**

Post-chemotherapy vs Pre-chemotherapy comparisons yielded 22 and 1 differentially expressed genes in the MRH and MRL groups, respectively. The number of genes differentially over-expressed in both MR groups was lower than in the case of GR and decreased as pathologic response to chemotherapy became poorer. On the other hand, the Her2G showed 30 genes differentially over-expressed and 2 genes differentially under-expressed after chemotherapy plus Trastuzumab, which is the closest result to the one observed in the GR group. With respect to the grade of homology between the gene sets differentially over-expressed in the different experimental groups, 93.3%, 90.9% and 100% of the genes differentially over-expressed in the Her2G, MRH and MRL groups were also differentially over-expressed in the GR group.

Pre-chemotherapy comparisons between groups defined by more similar responses to chemotherapy, as GR vs MRH and MRL vs BR, did not identify any significant gene differentially expressed between then, showing their similarities at the molecular level. On the other hand, we observed that the pre-chemotherapy comparison between GR vs MRL identified 49 genes significantly repressed in the GR group with respect the MRL group. The patients from MRL group are defined by a similar pathologic response to chemotherapy to those composing the BR group and, interestingly, the same comparison involving BR (GR vs BR before chemotherapy) yielded a similar result. Nevertheless, no statistically significant difference between MRH and BR groups were observed before chemotherapy. The comparisons between “edge-responses” and mid-responses showed that MRH retains a high proportion of the molecular characteristics of its closest group in terms of chemo-sensitivity and clinical aggressiveness, given that in the gene list resulting from the pre-chemotherapy comparison GR vs MRL we can find the repression of the 69,39% of the genes identified by the comparison GR vs BR.

Together, these results would indicate a concordance between similarities in the observed pathologic response to chemotherapy and the modulation of molecular mechanisms responsible for chemo-resistance. With respect to the Her2G group, pre-chemotherapy comparisons identified 4 genes significantly over-expressed with respect to the GR group and 2 repressed genes with respect to the BR group. We found no overlap between these sequences before, what may account for the known differences at the molecular level between Her2-positive tumors and Her2-negative tumors (19).

With respect to post-chemotherapy, comparison between MRH and MRL showed the greatest number of differentially expressed genes, highlighting the molecular heterogeneity of intermediate responses to chemotherapy. In the case of the Her2G, it behaved similar to GR group after chemotherapy, in contrast with the results of the same comparison before chemotherapy. As mentioned in the main document of this study, patients concurrently treated with Tastuzumab and anthraclycline and taxane-based chemotherapy frequently achieve good pathological responses to systemic treatment (20), similar to those that characterize our GR experimental group. Then, it seems reasonable to think that the observed gene expression similarities found between GR and Her2G after chemotherapy are a consequence of their similar response to treatment both at genetic and histological levels.
